# Interactions between ionizing radiation and *Vairimorpha (Nosema) ceranae* on the honeybee, *Apis mellifera L.*

**DOI:** 10.1371/journal.pone.0339853

**Published:** 2026-01-09

**Authors:** Margot Crevet, Béatrice Gagnaire, Jean-Marc Bonzom, Nicolas Dubourg, Michel Pélissier, Fabrice Daian, Gabriel Bon, Loïc Quevarec, Luc P. Belzunces, Jean-Luc Brunet

**Affiliations:** 1 Autorité de Sûreté Nucléaire et de Radioprotection, Laboratoire d’Ecologie et d’éCOtoxicologie des radionucléides, Saint Paul lez Durance, France; 2 Institut National de Recherche pour l’Agriculture, l’Alimentation et l’Environnement, Unité de Recherche 406 Abeilles and Environnement, Toxicologie Environnementale, Avignon, France; 3 Aix Marseille Université, Centre National de la Recherche Scientifique, Laboratoire Informatique et Systèmes, Marseille, France; 4 Aix Marseille Université, Laboratoire Informatique et Systèmes, Marseille, France; 5 Aix Marseille Université, Commissariat à l’Énergie Atomique et aux Énergies Alternatives, Centre National de la Recherche Scientifique, Institut de Biosciences et Biotechnologies d’Aix-Marseille, Luminy Génétique et Biophysique des Plantes, Marseille, France; 6 Laboratoire de Chimie Bactérienne, Institut de Microbiologie de la Méditerranée, Centre national de la recherche scientifique, Aix-Marseille Université, Marseille, France; Institute of Apicultural Research, CHINA

## Abstract

The global decline of honeybee colonies represents a major ecological concern, primarily attributed to simultaneous exposure to multiple stressors. These include biotic pressures, such as parasitic infections, and abiotic pressures, such as exposure to ionizing radiation, which remains poorly understood. Assessing their combined effects provides novel insights into how biological and radiological stressors interact within the organism. Here, we investigated the individual and combined effects of *Vairimorpha ceranae* (formerly *Nosema ceranae*) infection and chronic gamma irradiation (14 µGy/h or 14 × 10³ µGy/h) on honeybee health. Measurements included survival, syrup consumption, spore load, and biomarkers related to energy metabolism, antioxidant defenses, immunity, detoxification, and neural enzyme activity. Two successive experiments, conducted at different collection periods, allowed us to account for biological variability between bee cohorts. Infection by *V. ceranae* caused high mortality and major impairments in metabolic, antioxidant, and immune functions. Ionizing radiation induced more moderate effects, characterized by redox imbalance and reduced detoxification capacity, which varied with dose rate. Under combined exposure, the two stressors produced mainly antagonistic interactions affecting antioxidant, immune, and detoxification systems. However, a synergistic effect was observed on ATP production, suggesting an energetic compensation mechanism. These findings highlight complex physiological disturbances, revealing the multifactorial vulnerability of honeybees and emphasizing the need to integrate interactions between multiple stressors and natural biological variability into ecotoxicological assessments.

## Introduction

The honeybee (*Apis mellifera L.*) plays a crucial role in pollinating both agricultural and wild plants, sustaining biodiversity and syrup production [1]. However, global honeybee colony losses pose a significant threat to pollination and the beekeeping industry, primarily due to multiple stressors, including habitat loss, nutritional stress, pesticide exposure, climate change, and pathogenic organisms [2]. Among these pathogens, *Vairimorpha (*formerly *Nosema) ceranae* [3]*,* a microsporidian parasite, is one of the most prevalent and harmful. Its spores infect midgut epithelial cells, causing tissue damage that can lead to repeated self-infection and extensive cellular destruction, ultimately disrupting intestinal epithelial turnover [4]. The parasite also alters host metabolism, affecting carbohydrate and lipid pathways [4,5], which leads to energetic stress and increased syrup consumption [6,7]. *Vairimorpha ceranae* infection reduces immune gene expression [5,8,9] and induces oxidative stress [6,7,10], all of which compromise honeybee health, brood production, and colony survival [11]. Moreover, it interacts synergistically with pesticides, further weakening immunity and increasing mortality [7,12–14]. Understanding these multifactorial interactions remains crucial for mitigating colony decline.

Environmental contamination by ionizing radiation from nuclear activities or accidents may pose a further threat to honeybees and other insects, notably to survival, immunity, or reproduction [15–17]. The International Commission on Radiological Protection (ICRP) has identified eusocial bees as a reference animal (Reference Animal and Plant, RAP) for assessing the potential ecological effects of ionizing radiation on insects within its Derived Consideration Reference Level (DCRL) framework. This framework is based on the assumption that insects are relatively tolerant to ionizing radiation and that no significant biological effects are expected within and below 400–4000 µGy/h [18]. However, this threshold was established without experimental data on Hymenoptera, the group that includes bees and bumblebees. Several studies have since demonstrated biological effects at dose rates well below this threshold. For example, in *Apis mellifera*, chronic exposures to gamma radiation between 0.2 µGy/h and 24.5 mGy/h resulted in a decrease in antioxidant and immune markers [19]. Similarly, in bumblebees, exposures to 50 µGy/h led to reduced reproduction [17], while doses between 50 and 200 µGy/h were associated with disruptions of fundamental metabolic processes [20].

This study aimed to investigate how chronic exposure to ionizing radiation, at levels comparable to those found in contaminated environments following nuclear activities or accidents, affects honeybees already infected with *V. ceranae*. Newly emerged bees were infected with *V. ceranae* and subsequently exposed to continuous gamma radiation for 14 days. The objectives of this study were (i) to experimentally assess the potential interaction between ionizing radiation and *V. ceranae* infection on honeybee mortality, syrup consumption, and parasite virulence, and (ii) to examine how ionizing radiation affects key physiological functions in *V. ceranae*-infected bees, particularly their antioxidative defenses, immune response, and energy metabolism using biomarker measurements. By combining infection and irradiation under controlled laboratory conditions, this work aims to provide an integrated understanding of how biological and radiological stressors jointly influence honeybee health, thereby contributing to a broader assessment of the chronic effects of low-dose radiation on pollinator species.

## Materials and methods

### Honeybee management and protocol for *Vairimorpha ceranae* infection and gamma irradiation

Emerging *A. mellifera* honeybees (≤ 24 h old) were used for this experiment. In April (Experiment A) and May 2022 (Experiment B), a dozen brood-sealed frames were taken from five hives in the experimental apiary of the Bees & Environment research unit at INRAE Research center in Avignon, France. These frames were placed in an incubator at 34 ± 2°C and 60 ± 5% relative humidity for approximately 15 hours. Newly emerged honeybees were then collected directly from brood frames, pooled across frames, and randomly distributed into four to eight plastic cages (8 x 5 x 4 cm), with 50 honeybees per cage. Cages were then placed in a incubator in dark conditions at 30°C ± 2°C and 50 ± 5% relative humidity. Honeybees were fed *ad libitum* a sucrose syrup (60% w/v sucrose and 1% v/v bee food supplement; Provitabee, Apiculture Remuaux, Barbentane, France) for the duration of the study. They were also fed crushed organic pollen during the first three days. To maintain hygiene, a sheet of filter paper was placed at the bottom of each cage and replaced every two to three days.

The day after emergence, honeybees were collectively and orally infected with *V. ceranae*. Each honeybee received an average dose of 100,000 spores while feeding. To make the consumption of *V. ceranae* more attractive, bees were subjected to a 3-hour fast, and *Vairimorpha* spores were suspended in a 60% (w/v) sucrose syrup.

Three days after infection, the honeybees were exposed to gamma radiation. This exposure was continuous and lasted 14 days, starting on day 4. This duration was chosen because worker bees generally remain inside the hive for about two to three weeks before beginning to forage, making it a representative phase of their in-hive adult life and a suitable model to assess the effects of chronic exposure [21]. The external gamma radiation exposure was conducted at the Mini Irradiator for Radio Ecology ^137^Cs irradiation facilities of the “Nuclear Safety and Radiation Protection Authority” (MIRE, ASNR, Cadarache, France). Two incubators (Panasonic MIR-154-PE), each equipped with a solid source of ^137^Cs (activity of 1.64 GBq as of May 16, 2022), were used for exposure. Honeybees were either exposed to both ionizing radiation and *V. ceranae* infection*,* only ionizing radiation, or only *V. ceranae* infection. Control bees were neither infected nor irradiated. Cages containing 50 honeybees were arranged equidistantly around the radiation source. Glass RPL (RadioPhotoLuminescence) dosimeters (GD-300 series; Chiyoada Technologies, Japan) were positioned on the cage faces nearest and farthest from the source to measure the exact dose rate received by the honeybees.

Two experiments (A and B) were conducted. Honeybees for Experiment A were collected in April, and those for Experiment B in May, allowing us to evaluate how initial physiological differences related to the collection period influenced the measured parameters. In Experiment A, a low dose rate of 14 µGy/h and a high dose rate of 14 × 10³ µGy/h were used. A single honeybee sample was taken at day 18, 14 days of irradiation, for spore count and biomarker analysis. In Experiment B, only the high dose rate (14 × 10³ µGy/h) was tested, and several time-dependent honeybee samples were taken at days 4, 8, 12, and 18 (corresponding to 0, 2, 8, and 14 days of irradiation). The low dose rate of 14 µGy/h is close to the reference threshold of 10 µGy/h established by Garnier-Laplace et al. [22], below which 95% of ecosystems are considered protected from the effects of ionizing radiation. The 14 µGy/h value is also below the lower band (400 µGy/h) of the DCRL, which assumes there are no harmful effects on insects [18]. In contrast, the high dose rate of 14 × 10³ µGy/h is used to explore the mechanisms of action at rates exceeding those found in the environment after a nuclear accident. The 14 × 10³ µGy/h value is also well above the upper band of the DCRL for bees at 4 mGy/h, thus falling within the dose rate range for which proven effects have been observed [18]. This choice was made to focus on the potential cumulative effects of prolonged exposure at a biologically relevant high dose, as well as to maximize the likelihood of detecting time-dependent physiological and molecular responses to radiation that could occur soon after a nuclear accident.

### Parameters assessed

#### Syrup consumption and mortality.

Syrup consumption and mortality were recorded every two to three days until the end of the experiment. Dead honeybees were consistently counted and removed from cages. Individual syrup consumption was assessed by measuring the weight of food tubes and adjusting for the number of surviving honeybees. Each cage was equipped with two 5 mL feeders containing a sucrose syrup solution. Syrup consumption and mortality were determined after 0, 2, 4, 7, 9, 11, and 14 days of irradiation at 14 µGy/h and 14 × 10³ µGy/h in Experiment A, and determined after 0, 2, 4, 6, 8, 11, and 14 days of irradiation at 14 × 10³ µGy/h in Experiment B.

#### Counting of *V. ceranae* spores.

The number of *V. ceranae* spores was determined using 24 honeybees per experimental modality. From each cage, 8–10 abdomens were collected to prepare a tissue extract for quantifying the number of *V. ceranae* spores. Abdomens were injected with 4 mL of distilled water and placed in Bioreba extraction bags before being homogenized using a ball-bearing. Spore concentration in the homogenates was determined by counting the number of spores in Malassez cells under a light microscope. Five Malassez cell deposits were required for each homogenate and photos were taken and analyzed using Artificial Intelligence (AI) to count the number of spores per image (S1 Text).

#### Physiological effects, tissue homogenization, and biomarker analysis.

Physiological markers were analyzed in bees that survived 14 days of irradiation exposure (four cages per modality) from Experiment A and 0, 2, 4, 8, and 14 days from Experiment B. To determine the kinetics of biomarker modulation, two cages per modality were taken at each time point (a total of 10 cages for the irradiation controls and eight cages for the other modalities). Prior to tissue sampling, the honeybees were first anesthetized with CO_2_. The head was then immediately separated from the thorax, and the midgut was removed from the abdomen. The heads, abdomen (without the intestinal tract), and midgut were then placed in 2 mL Eppendorf tubes, weighed, and immediately stored at −20°C until analysis. For each treatment, replicates (n = 6 samples) of pooled tissues from three honeybees were analyzed. Each sample was assayed in triplicate using a UV-Visible microplate spectrophotometer (Bioteck). Tissues were homogenized using a TissueLyser II high-speed homogenizer (Qiagen) for five 10-second cycles, with 30-second intervals between cycles. Homogenization was performed in an extraction medium consisting of 10 mM sodium chloride, 1% w/v Triton X-100, 40 mM sodium phosphate (pH 7.4), and protease inhibitors (2 μg/mL pepstatin A, leupeptin, and aprotinin, 0.1 mg/mL soybean trypsin inhibitor) to produce 10% w/v tissue extracts [23]. After homogenization, the extracts were centrifuged at 15,000 *g* for 22 minutes at 4°C, and their supernatants were stored on ice prior to assay.

By incorporating various biomarkers, we aimed to measure a wide range of physiological traits necessary to obtain a comprehensive understanding of the potential effects of ionizing radiation on antioxidant, detoxification, metabolic, immune, and neural functions that are indicated in Table 1. The concentration of Acetylcholinesterase (AChE) was measured at an absorbance of λ = 412 nm, following Ellman et al. [24] and modified by Belzunces et al. [23]. Glutathione peroxidase (GP) was assayed by monitoring the conversion of nicotinamide adenine dinucleotide phosphate (NADPH) to nicotinamide adenine dinucleotide phosphate (NADP^+^) at 340 nm [25]. Glutathione reductase (GR) was also monitored at 340 nm by the conversion of NADPH to NADP^+^ [25]. Glutathione-S-transferase (GST) was determined by measuring the conjugation of reduced glutathione (GSH) to 1-chloro-2,4-dinitrobenzene (CDNB) at 340 nm [26]. Superoxide dismutase (SOD) was indirectly measured by the generation of reduced nitro blue tetrazolium (NBT), which was monitored at 560 nm [27]. Catalase (CAT) was assayed by measuring the decomposition of hydrogen peroxide (H2O2) at 240 nm [28]. Glucose-6-phosphate dehydrogenase (G6PDH) was determined by monitoring NADPH formation at 340 nm [29]. Phenol oxidase (POX) was determined by monitoring the conversion of 3,4-dihydroxy-L-dihydroxyphenylalanine (L-DOPA) to melanin at 490 nm [12]. Alkaline phosphatase (ALP) was assessed by conversion of p-nitrophenyl phosphate (p-NPP) to p-nitrophenol at 410 nm [30]. Lactate dehydrogenase (LDH) was determined by regenerating nicotinamide adenine dinucleotide (NAD^+^) at 340 nm [31]. Triglyceride (TG) was determined using Triglycerides Colorimetric Assay Cayman kit (Bertin). Absorbance was measured at a wavelength of 550 nm. Glyceraldehyde-3-phosphate dehydrogenase (GaPDH) was measured by NADH oxidation followed at 340 nm [29]. Adenosine triphosphate (ATP) was determined using the ATP^lite^ kit (Revvity) based on luminescence measurements generated by the oxidation of D-luciferin by luciferase, following Ben Abdelkader et al. [27]. The activity of carboxylesterases (CaE)1 was measured at 568 nm, and CaE3 was measured at 410 nm [31]. All products used were manufactured by Sigma-Aldrich (Saint-Quentin-Fallavier, France).

**Table 1:** Employed biomarkers and their targeted biological functions in tissues (Experiments A and B).

Biomarker	Tissue	Functions	Experiment	
			A	B
GP	Midgut	Antioxidant defenses	X	
GR	Midgut	Antioxidant defenses	X	
SOD	Head	Antioxidant defenses		X
CAT	Head	Antioxidant defenses/ Detoxification		X
GST	Head	Antioxidant defenses/ Detoxification/ Metabolism		X
G6PDH	Head	Antioxidant defenses/ Metabolism	X	
G6PDH	Abdomen	Antioxidant defenses/ Metabolism		X
GaPDH	Head	Metabolism	X	
GaPDH	Abdomen	Metabolism		X
ATP	Head	Metabolism	X	
ATP	Abdomen	Metabolism		X
LDH	Head	Metabolism	X	
TG	Abdomen	Metabolism	X	
POX	Abdomen	Immunity	X	X
ALP	Midgut	Immunity	X	X
CaE1	Midgut	Detoxification/ Metabolism/ Immunity	X	
CaE1	Abdomen	Detoxification/ Metabolism/ Immunity	X	X
CaE3	Head	Detoxification/ Metabolism/ Immunity	X	
CaE3	Abdomen	Detoxification/ Metabolism/ Immunity	X	X
AChE	Head	Neural activity		X

### Statistical analyses

All statistical analyses were performed using R (version 4.2.1). Analyses were performed separately for the two experimental setups, applying statistical methods tailored to the nature of the measured variables and data distributions. The effects of the Experiment A conditions on biomarker responses and pathogen load were assessed using generalized linear models (GLM), which were adjusted according to the distribution of the response variables. Model assumptions were verified by a residual analysis, Shapiro test for normality, and Levene’s test for homogeneity of variances (using the car package [32]). In cases where an assumption was violated, a logarithmic or Box-Cox transformation was applied to the data. Multiple comparisons between groups were performed using Tukey’s post-hoc test via the glht() function in the multcomp package [33], allowing for the evaluation of significant differences between modalities. For biomarker and pathogen load analyses in Experiment B, as well as for the mortality rate and syrup consumption across both experiments, the data were analyzed using generalized additive mixed-effects models (GAMM) with the gamm4 package [34]. This approach was chosen to model nonlinear trends over time while incorporating random effects when necessary. Models for biomarker responses and pathogen load were fitted using a gamma distribution with a logarithmic link function, whereas mortality was modelled using a binomial distribution with a logit link function (cbind(dead, alive)). To account for time-dependent variation in biological responses, we incorporated modality-specific splines (s(day, by = modality, k = 5)). These models enable a visual assessment of potential synergistic or antagonistic effects between treatment groups over time, as evidenced by differences in curve shape or dynamics. Predictions from the GAMM models were visualized as smoothed curves with 95% confidence intervals to illustrate trends over time, while the results obtained from the GLM were represented using boxplots. All figures were generated with ggplot2 [35], and figure combinations were performed using the patchwork package. All analyses were conducted with a significance threshold of α = 0.05. The interaction between irradiation and infection was evaluated for all measured parameters by including an interaction term (gamma*Vairimorpha) in the GAMM and GLM models. The FactoMineR package [36] was used for Principal Component Analysis (PCA). Prior to performing the PCA, missing data were imputed using the imputePCA method from the missMDA package [37], which provides a missing data imputation consistent with the PCA framework. The optimal number of dimensions to retain for imputation was evaluated using the estim ncpPCA function.

## Results

### Effects of ionizing radiation and *V. ceranae* infection on spore count, mortality rate, and syrup consumption

Spore load, mortality, and syrup consumption were measured to assess the impact of *Vairimorpha ceranae* infection and/or gamma irradiation on bees. Infection elevated spore count, increased bee mortality, and disrupted syrup consumption. In Experiment B, irradiation (14 × 10³ µGy/h) increased mortality and decreased consumption. When combined with high-dose-rate irradiation, *V. ceranae* infection produced an antagonistic effect on consumption (also only observed in Experiment B).

**Fig 1:**
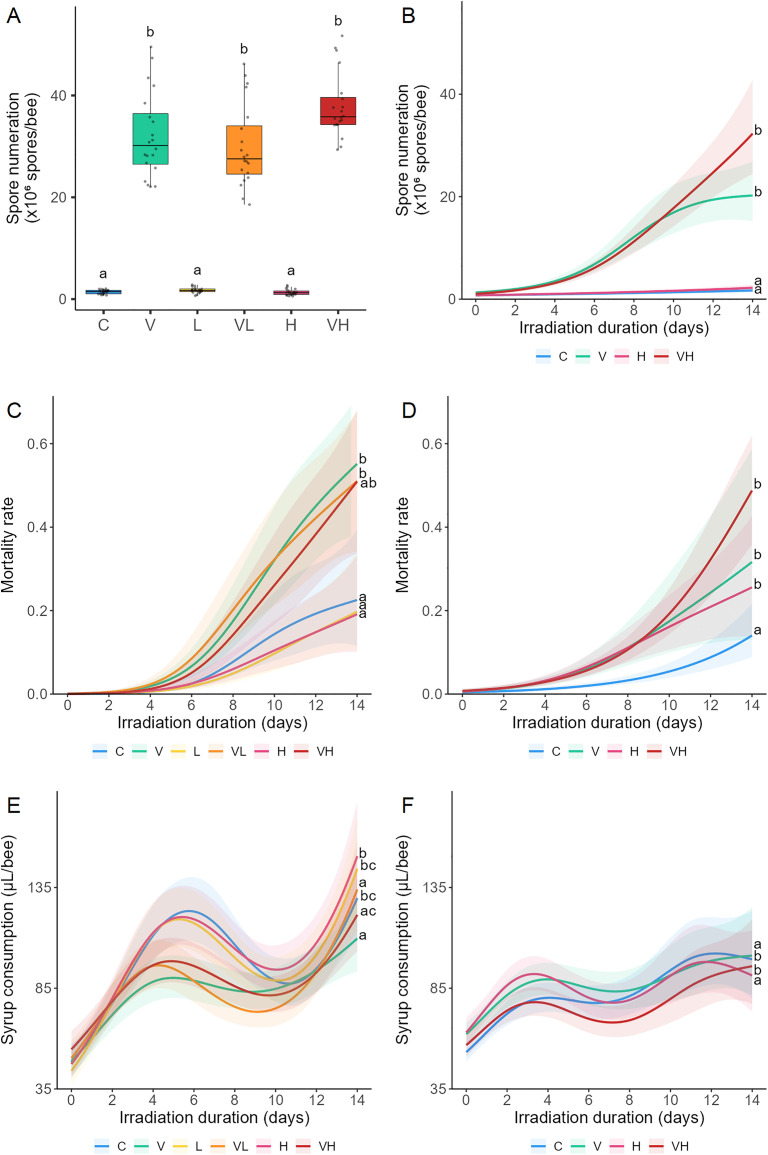
Effects of ionizing radiation and *Vairimorpha ceranae* infection on spore numbers, mortality, and syrup consumption. Bees were infected with *V. ceranae* one day after emerging, and three days after infection, they were continuously exposed to gamma radiation for 14 days. C: Control bees, neither irradiated nor infected. V: Bees are only infected. L: Bees are only irradiated at 14 µGy/h. VL: Bees are both infected and irradiated at 14 µGy/h. H: Bees are only irradiated at 14 × 10³ µGy/h. VH: Bees are both infected and irradiated at 14 × 10³ µGy/h. Measurements were taken after 14 days of irradiation in Experiment A and during 14 days in Experiment B for spore numbers (A and B, respectively), Mortality rate (C and D, respectively), and Syrup consumption (E and F, respectively). Data represent the average number of spores/bee ± SD (Experiment A) or ± CI95 (Experiment **B)**, the average mortality rate ± CI95, and the average syrup consumption per µL/bee ± IC95. Data with different letters are significantly different (p < 0.05).

Significant differences among the different modalities (C, V, L, VL, H, VH; with L and VL assessed only in Experiment A – see Fig 1 legend for details) were evaluated for each parameter, including spore count, mortality rate, and syrup consumption (Fig 1; S1 Table and S2 Table). In both experiments, spore count was significantly higher in honeybees infected with *V. ceranae* (V, VL, VH) than in uninfected honeybees (C, L, H). Spore count did not differ significantly between uninfected bees (C, L, H), nor between infected bees (V, VL, VH) (Fig 1A and 1B). For the mortality rate in Experiment A, significantly higher mortality was observed in infected bees (V, VL) compared to uninfected bees (C, L, H) (Fig 1C). In Experiment B, significantly higher mortality was observed in modalities V, H, and VH compared to C (Fig 1D). For syrup consumption in Experiment A, significantly lower consumption was observed in the infected bees (V, VL, VH) compared to the uninfected bees (C, L, H) (Fig 1E). In Experiment B, significantly lower syrup consumption was observed in the uninfected bees (C, H) compared to the infected bees (V, VH). Moreover, significantly lower syrup consumption was observed in the irradiated bees (H, VH) compared to the unirradiated bees (C, V) (Fig 1F).

In Experiment B, across all modalities (C, V, L, VL, H, VH), all three endpoints exhibited significant temporal effects: spore count and mortality increased over time, and syrup consumption varied over time (Fig 1, Table 2). Furthermore, no significant interaction between irradiation and *V. ceranae* was observed for spore count (Experiment A and B), mortality (Experiment A and B), and consumption (Experiment A) (Table 3 and Table 4). However, a significant interaction was observed for consumption in Experiment B, where an antagonistic interaction was observed for VH (Table 4).

**Table 2:** Effects of ionizing radiation and *Vairimorpha ceranae* infection on spore numbers, syrup consumption, mortality rate, and biomarkers.

Experiment	Parameters	C	V	L	VL	H	VH
A	Mortality	***	***	***	***	***	***
B	Mortality	***	***	na	na	***	***
A	Consumption	***	***	***	***	***	***
B	Consumption	***	***	na	na	***	***
B	Spores number	***	***	na	na	***	***
B	SOD activity	ns	ns	na	na	ns	*
B	CAT activity	**	***	na	na	***	***
B	GST activity	**	*	na	na	ns	ns
B	G6PDH activity	ns	ns	na	na	ns	***
B	GaPDH activity	ns	*	na	na	ns	***
B	ATP level	ns	*	na	na	ns	ns
B	POX activity	ns	ns	na	na	***	***
B	ALP activity	ns	ns	na	na	ns	***
B	CaE 1 activity	*	***	na	na	***	***
B	CaE 3 activity	ns	ns	na	na	***	***
B	AChE activity	ns	ns	na	na	ns	*

Data represent the p-values resulting from a generalized additive mixed model analysis to assess the temporal effect of different parameters during 14 days. C: Control bees, neither irradiated nor infected; V: Bees are only infected; L: Bees are only irradiated at 14 µGy/h; VL: Bees are both infected and irradiated at 14 µGy/h; H: Bees are only irradiated at 14 × 10³ µGy/h; VH: Bees are both infected and irradiated at 14 × 10³ µGy/h. Parameters analyzed included spore number in Experiment B, syrup consumption, mortality in both experiments (A and B), and biomarkers in Experiment B. Results show significant effects for all factors tested, with significance levels indicated as follows: (*): p < 0.05; (**): p < 0.01; (***): p < 0.001; ns: not significant; na: not available.

**Table 3:** Effects of irradiation and infection, considered individually and in interaction, on the parameters studied in Experiment A.

Parameters	Significant effects of the individual stressors			Significant effect and characterization of the interaction between stressors.	
	V	L	H	VL	VH
Mortality	***	ns	ns	ns	ns
Consumption	**	ns	ns	ns	ns
Spore number	***	ns	ns	ns	ns
Midgut GP	ns	ns	ns	ns	ns
Midgut GR	ns	ns	ns	ns	ns
Head G6PDH	ns	ns	ns	ns	ns
Head GaPDH	ns	ns	*	ns	ns
Head ATP	*	ns	ns	ns	ns
Head LDH	ns	ns	ns	* Antagonistic	ns
Abdomen TG	ns	ns	ns	ns	ns
Abdomen POX	ns	ns	ns	ns	ns
Midgut ALP	ns	***	ns	ns	ns
Midgut CaE1	ns	***	*	ns	* Antagonistic
Abdomen CaE1	ns	ns	ns	** Antagonistic	ns
Midgut CaE3	**	**	***	ns	*** Antagonistic
Abdomen CaE3	*	**	ns	** Antagonistic	ns

The table summarizes the significant effects of irradiation and infection, considered individually and in interaction, on all tested parameters, as well as the characterization of said interaction. An absence of interaction characterization indicates that no interaction was detected. The analyzed parameters included spore number, syrup consumption, mortality, and biomarkers. C: Control bees, neither irradiated nor infected; V: Bees are only infected; L: Bees are only irradiated at 14 µGy/h; H: Bees are only irradiated at 14 × 10³ µGy/h; VL: Bees are both infected and irradiated at 14 µGy/h; VH: Bees are both infected and irradiated at 14 × 10³ µGy/h. Significance levels are indicated as follows: (*): p < 0.05; (**): p < 0.01; (***): p < 0.001; ns: not significant.

**Table 4:** Effects of irradiation and infection, considered individually and in interaction, on the parameters studied in Experiment B.

Parameters	Significant effects of the individual stressors		Significant effect and characterization of the interaction between stressors.
	V	H	VH
Mortality	**	**	ns
Consumption	*	**	*** Antagonistic
Spore number	***	ns	ns
Head SOD	*	ns	* Antagonistic
Head CAT	ns	ns	ns
Head GST	ns	ns	* Antagonistic
Abdomen G6PDH	***	***	*** Antagonistic
Abdomen GaPDH	ns	ns	ns
Abdomen ATP	ns	ns	* Synergistic
Abdomen POX	***	***	*** Antagonistic
Midgut ALP	*	**	** Antagonistic
Abdomen CaE1	***	***	*** Antagonistic
Abdomen CaE3	***	ns	*** Antagonistic
Head AChE	ns	ns	ns

The table summarizes the significant effects of irradiation and infection, considered individually and in interaction, on all tested parameters, as well as the characterization of said interaction. An absence of interaction characterization indicates that no interaction was detected. The analyzed parameters included spore number, syrup consumption, mortality, and biomarkers. C: Control bees, neither irradiated nor infected; V: Bees are only infected; H: Bees are only irradiated at 14 × 10³ µGy/h; VH: Bees are both infected and irradiated at 14 × 10³ µGy/h. Significance levels are indicated as follows: (*): p < 0.05; (**): p < 0.01; (***): p < 0.001; ns: not significant.

### Physiological effects of ionizing radiation and *V. ceranae* infection

To characterize the physiological impact of *Vairimorpha ceranae* infection and/or gamma irradiation on honeybees, several biomarkers were analyzed. Biomarker analyses were conducted to target several biological functions of interest, including oxidative stress defenses, metabolic integrity, immune defenses, and neural activity.

#### Effects of ionizing radiation and *V. ceranae* infection on honeybee defense against oxidative stress and on metabolism.

Antioxidant activity was assessed by measuring GP and GR in the midgut and G6PDH in the head in Experiment A, and by measuring SOD, CAT, and GST in the head and G6PDH in the abdomen in Experiment B. Modifications in antioxidant activity occurred exclusively in Experiment B. Infection altered SOD, CAT, and G6PDH, irradiated affected G6PDH, and combined exposure influenced CAT, G6PDH, SOD, and GST. Furthermore, the combination of infection and irradiation revealed antagonistic interactions for SOD, GST, and G6PDH.

**Fig 2:**
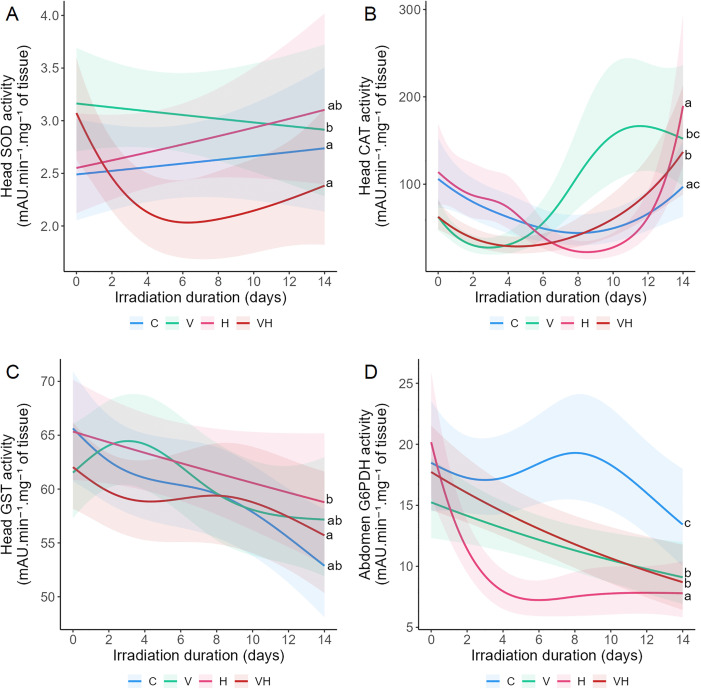
Effects of ionizing radiation and *Vairimorpha ceranae* infection on biomarkers of antioxidative defenses. Bees were infected with *V. ceranae* one day after emerging, and three days after infection, they were continuously exposed to gamma radiation for 14 days. C: Control bees, neither irradiated nor infected. V: Bees are only infected. H: Bees are only irradiated at 14 × 10³ µGy/h. VH: Bees are both infected and irradiated at 14 × 10³ µGy/h. Head SOD activity **(A)**, head CAT activity **(B)**, head GST activity **(C)**, and abdomen G6PDH activity (D) were measured during 14 days in Experiment **B.** Data represent the mean activity of six head or abdomen extracts tested in triplicate ± CI95 per cage (2 cages/modality/day). Data are expressed as milli Absorbance Units **(mA)**.min^-1^.mg^-1^ of tissue. Data with different letters are significantly different (p < 0.05).

Tests of significance between the different modalities for each biomarker were measured (Fig 2, S2 Table), whereby no significant effects were observed in Experiment A between modalities for GP, GR, and G6PDH (S1 Fig and S1 Table). On the contrary, Experiment B showed that SOD was significantly higher in V compared to both C and VH (Fig 2A). In addition, SOD decreased over time in VH, with a sharp drop during the initial days (until day 5), followed by a slight increase (Fig 2A, Table 2). CAT was significantly higher in VH compared to C, and H differed significantly from both V and NH (Fig 2B). In addition, CAT exhibited a biphasic profile across all modalities, characterized by an initial drop that was followed by a gradual increase over time. In C and H, the decrease in CAT persists until day 9. This decrease period is shorter (until around day 4–5) in V and VH, with the ascending phase observed earlier than what was observed in C and H (Fig 2B, Table 2). GST in VH was significantly lower than in H (Fig 2C). GST kinetics in C and V showed a decrease over time (Fig 2C, Table 2). G6PDH was significantly reduced in V, VH, and H compared to C. Furthermore, G6PDH was also reduced in H compared to V and VH (Fig 2D). G6PDH kinetics in VH exhibited a decrease over time (Fig 2D, Table 2). The results also reflected an antagonistic interaction between V and H for SOD, GST, and G6PDH in Experiment B (Table 4).

Metabolic status was assessed by measuring GaPDH, ATP, and LDH in the head and TG in the abdomen in Experiment A. In Experiment B, GaPDH and ATP were additionally measured in the abdomen. Differences in metabolic activity were observed between irradiated and infected bees for GaPDH in both experiments and for ATP in Experiment A. Infection alone led to a progressive decline in GaPDH and ATP activity. However, bees exposed to both infection and irradiation simultaneously exhibited elevated ATP activity, reflecting a synergistic interaction between these stressors.

**Fig 3:**
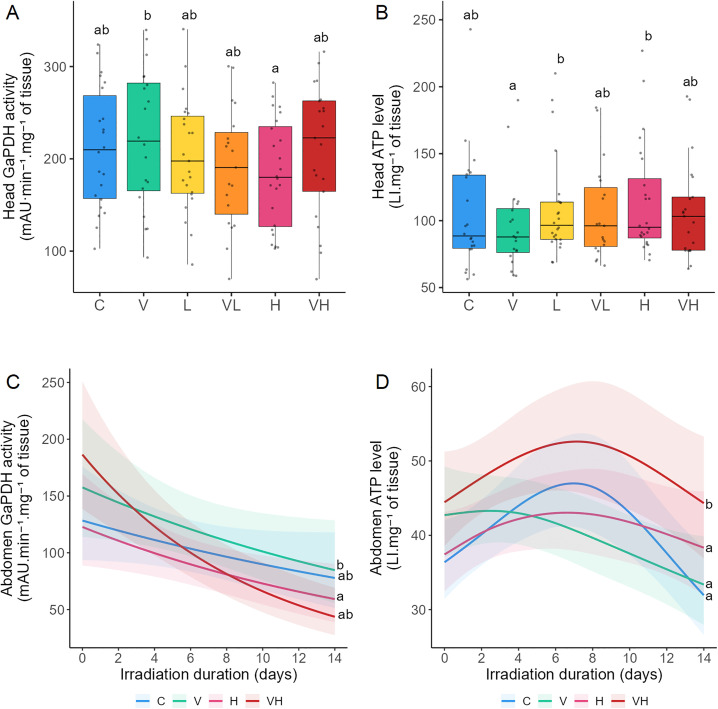
Effects of ionizing radiation and *Vairimorpha ceranae* infection on energy metabolism. Bees were infected with *V. ceranae* one day after emerging, and three days after infection, they were continuously exposed to gamma radiation for 14 days. C: Control bees, neither irradiated nor infected. V: Bees are only infected. L: Bees are only irradiated at 14 µGy/h. VL: Bees are both infected and irradiated at 14 µGy/h. H: Bees are only irradiated at 14 × 10³ µGy/h. VH: Bees are both infected and irradiated at 14 × 10³ µGy/h. Head GaPDH activity (A) and head ATP content (B) were measured after 14 days of irradiation in Experiment **A.** Abdomen GaPDH activity (C) and abdomen ATP content (D) were measured during 14 days in Experiment **B.** For Experiment A, the data represent the mean activity from six head extracts tested in triplicate ± SD per cage (4 cages/modality). For Experiment B, the data represent the mean activity from six abdomen extracts tested in triplicate ± 95% CI per cage (2 cages/modality/day). GaPDH activity is expressed as milli Absorbance Units **(mA)**.min^-1^.mg^-1^ of tissue, and ATP content is expressed as Luminescence Intensity.mg^-1^ of tissue. Data with different letters are significantly different (p < 0.05).

Tests of significance between the different modalities for each biomarker were conducted (Fig 3, S1 Table and S2 Table). In Experiment A, GaPDH was significantly higher in V compared with H (Fig 3A). Moreover, ATP was significantly reduced in V compared to L and H (Fig 3B). No significant effects were observed between modalities for LDH and TG (S1 Fig, S1 Table). In Experiment B, GaPDH was significantly higher in V compared with H (Fig 3C). GaPDH kinetics showed a decrease over time for V and VH (Fig 3C, Table 2). ATP were significantly higher in VH than in C, V and H (Fig 3D). The kinetics of ATP exhibited a decrease in V over time (Fig 3D, Table 2). The results reflected a synergistic interaction between V and H for ATP in Experiment B (Table 2 and Table 3).

#### Effects of ionizing radiation and *V. ceranae* infection on immune defenses and neural activity.

In Experiments A and B, two markers involved in the immune defenses (POX and ALP) were investigated. POX and ALP were analyzed in the abdomen and midgut of bees, respectively. AChE, a neural enzyme, was also measured in the head in Experiment B. Immune function was altered in response to both infection and irradiation, as evidenced by a decrease POX activity and increase ALP activity (Experiment B). ALP was elevated at low radiation doses in Experiment A. An antagonistic interaction between infection and radiation was observed for POX and ALP (Experiment B), while no significant interaction was found for AChE.

**Fig 4:**
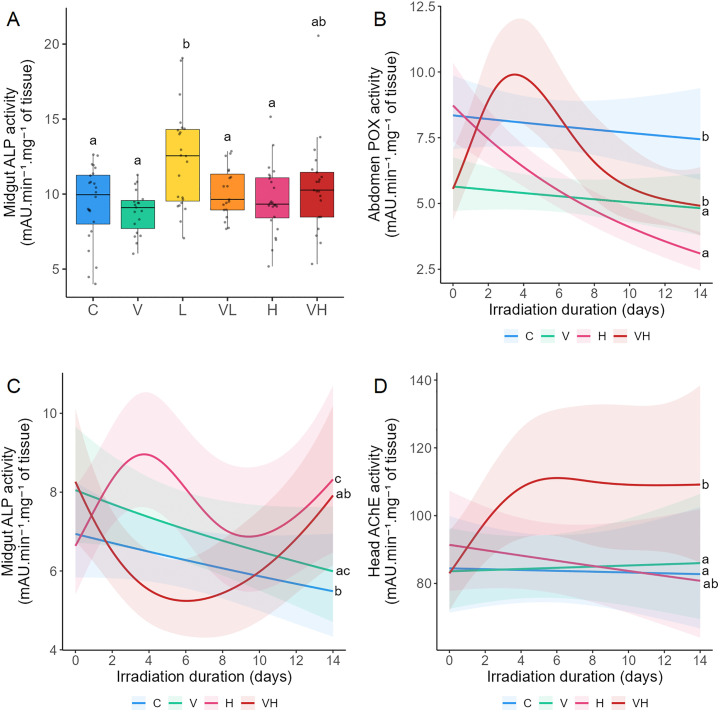
Effects of ionizing radiation and *Vairimorpha ceranae* infection on immune defenses and neural activity. Bees were infected with *V. ceranae* one day after emerging, and three days after infection, they were continuously exposed to gamma radiation for 14 days. C: Control bees, neither irradiated nor infected. V: Bees are only infected. L: Bees are only irradiated at 14 µGy/h. VL: Bees are both infected and irradiated at 14 µGy/h. H: Bees are only irradiated at 14 × 10³ µGy/h. VH: Bees are both infected and irradiated at 14 × 10³ µGy/h. Midgut ALP activity (A) was measured after 14 days of irradiation in Experiment **A.** Abdomen POX activity **(B)**, midgut ALP activity (C) and head AChE activity (D) were measured during 14 days in Experiment **B.** For Experiment A, the data represent the mean activity from six midgut extracts tested in triplicate ± SD per cage (4 cages/modality). For Experiment B, the data represent the mean activity from six abdomen, midgut, or head extracts tested in triplicate ± 95% CI per cage (2 cages/modality/day). Data are expressed as milli Absorbance Units **(mA)**.min^-1^.mg^-1^ of tissue. Data with different letters are significantly different (p < 0.05).

Tests of significance between the different modalities for each biomarker were conducted (Fig 4, S1 Table and S2 Table). In Experiment A, POX exhibited no significant differences across modalities (S1 Fig, S1 Table), and ALP was significantly elevated in L compared to C, V, VL, and H (Fig 4A). In Experiment B, POX in V and H was significantly reduced compared to C and NH (Fig 4B). Furthermore, POX kinetics showed a decrease over time for H, whereas in VH, POX increased sharply until four days of irradiation and then decreased significantly (Fig 4B, Table 2). ALP was significantly higher in V and H compared to C, and was higher in H compared to VH (Fig 4C). For VH, ALP kinetics decreased sharply until the 6th day of irradiation before returning close to its initial activity at time 0 (Fig 4C, Table 2). The results also indicated no interaction between infection and irradiation for ALP and POX in Experiment A (Table 3), whereas in Experiment B, an antagonistic interaction was observed for both activities (Table 4). AChE was significantly higher in VH compared to C and V (Fig 4C). Furthermore, in VH, AChE increased sharply up to day 4, followed by stagnation (Fig 4C, Table 2). The results also indicated no interaction between infection and irradiation for AChE (Table 4).

#### Effects of ionizing radiation and *V. ceranae* infection on carboxylesterases.

CaE are involved in detoxification, metabolism and immune defenses. CAE1 and 3 were analyzed in the midgut in Experiment A and in the abdomen in Experiments A and B. CaE activity decreased in bees that were only infected or irradiated, even at low dose rates. Antagonistic interactions between infection and irradiation were observed in the midgut and abdomen for CaE1 and 3.

**Fig 5:**
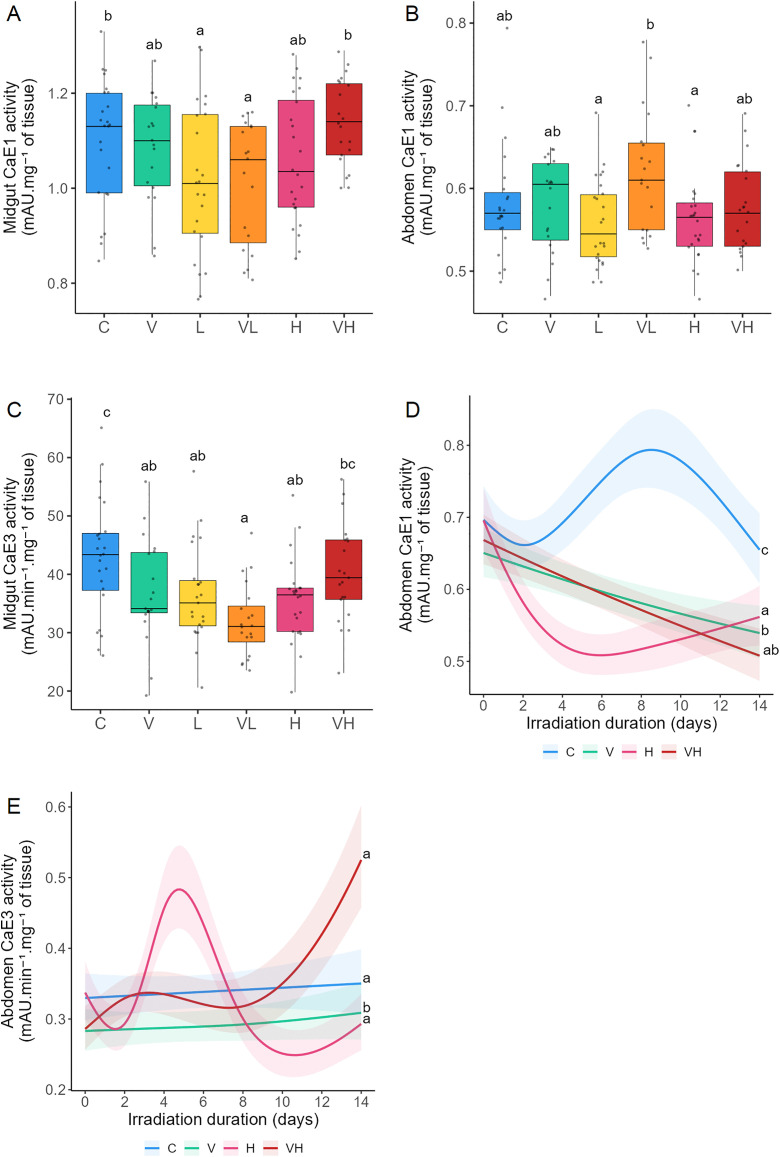
Effects of ionizing radiation and *Vairimorpha ceranae* infection on carboxylesterases. Bees were infected with *V. ceranae* one day after emerging, and three days after infection, they were continuously exposed to gamma radiation for 14 days. C: Control bees, neither irradiated nor infected. V: Bees are only infected. L: Bees are only irradiated at 14 µGy/h. VL: Bees are both infected and irradiated at 14 µGy/h. H: Bees are only irradiated at 14 × 10³ µGy/h. VH: Bees are both infected and irradiated at 14 × 10³ µGy/h. Midgut (A) and abdomen CaE1 activities **(B)**, and midgut CaE3 activity (C) were measured after 14 days of irradiation in Experiment **A.** Abdomen CaE1 activity (D) and abdomen CaE3 activity (E) were measured during 14 days in Experiment **B.** For Experiment A, the data represent the mean activity from six midgut or abdomen extracts tested in triplicate ± SD per cage (4 cages/modality). For Experiment B, the data represent the mean activity from six abdomen extracts tested in triplicate ± 95% CI per cage (2 cages/modality/day). CaE1 activity is expressed as milli Absorbance Units.mg^-1^ of tissue, and CaE3 activity is expressed as milli Absorbance Units **(mA)**.min^-1^.mg^-1^ of tissue. Data with different letters are significantly different (p < 0.05).

Tests of significance between the different modalities for each biomarker were measured (Fig 5, S1 Table and S2 Table). In Experiment A, CaE1 in the midgut was significantly reduced in both L and VL compared to both C and NH (Fig 5A). CaE1 in the abdomen in VL was significantly higher than in L and H (Fig 5B). Compared to C, CaE3 in the midgut underwent a significant decrease in V, L, VL, and H. CaE3 in VH was significantly higher than in NL (Fig 5C). In the abdomen, no significant effects were observed between modalities for CaE3 (S1 Fig, S1 Table). In Experiment B, CaE1 was lower in N, H, and NH than in C. CaE1 was also significantly different between V and H (Fig 5D). CaE1 activity exhibited a triphasic pattern for C, with a slight initial decrease, followed by an increase, and then a return to near day 0 levels. In contrast, CaE1 kinetics showed a marked decline followed by a gradual recovery in H, while in V and VH it decreased steadily over time (Fig 5D, Table 2). CaE3 was lower in V than in C, H, and VH (Fig 5E). CaE3 kinetics displayed triphasic and quadriphasic profiles for VH and H, respectively. In Experiment A, antagonistic interactions were detected for CaE1 and 3 in the abdomen between L and V, as well as for CaE1 and 3 in the midgut between V and H. In Experiment B, an antagonistic interaction between V and H was also observed for CaE 1 and 3 in the abdomen.

### Multivariate analysis on biomarkers

**Fig 6:**
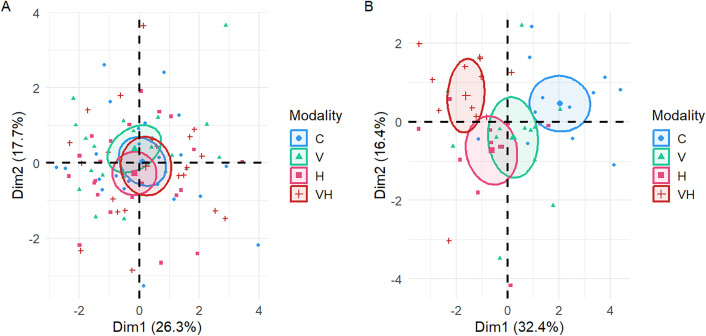
Principal Component Analysis (PCA) of biomarkers measured after 14 days of irradiation. PCA results for Experiment A (A) and B **(B)**. The points represent individual values, colored according to their experimental modality: C: Control bees, neither irradiated nor infected; V: Bees are only infected; H: Bees are only irradiated at 14 × 10³ µGy/h; VH: Bees are both infected and irradiated at 14 × 10³ µGy/h. The 95% confidence ellipses illustrate the dispersion of each group.

In Experiment A, the first two principal components (Dim1 = 26.3% and Dim2 = 17.7% of the variance) accounted for approximately 44% of the total data variability. Visually, the 95% confidence ellipses associated with each group partially overlapped, indicating intra-group variability and a moderate separation between modalities. The control group (C) was generally located near the center or slightly negative on the Dim1 axis. The infected group (N) appeared more dispersed, occupying both positions close to the control and more peripheral areas. The irradiated group (H) and the combined group (HN) were also found in a relatively central zone, overlapping slightly with the other modalities (Fig 6A). In Experiment B, the first two principal components (Dim1 = 32.4% and Dim2 = 16.4%) explained nearly half of the total variance. Here, the separation between groups was markedly more pronounced. The control group (C), clustered on the right side of the plot (positive Dim1), was clearly distinguishable from the area occupied by the other modalities. The infected group (N) was positioned closer to the center (with Dim1 values near zero). The irradiated group (H) was located predominantly in the lower region of the plot (negative Dim2). In contrast, the combined group (HN) was positioned higher (positive Dim2) and tended toward negative Dim1 values, placing it further from the control group (Fig 6B).

## Discussion

This study aimed to determine the combined impacts of *Vairimorpha ceranae* infection and chronic exposure to gamma ionizing radiation (14 µGy/h or 14 × 10³ µGy/h) on the physiology of the honeybee, *Apis mellifera L*. The evaluation focused on infection dynamics (spore count), mortality, syrup consumption and a range of biomarkers related to immune defenses, oxidative stress defenses, energy metabolism and neural activity.

The contrast observed between Experiments A and B, across the different analyses and for the same treatment modalities (infected bees, irradiated bees (14 × 10³ µGy/h), and irradiated + infected bees), suggests that bees in Experiment B were more strongly affected by the experimental stressors than those in Experiment A. Indeed, multivariate analyses of biomarker profiles showed that in Experiment A, some markers allowed only partial discrimination between conditions, resulting in substantial overlap among profiles. In contrast, in Experiment B, bees appeared to be more sensitive or more specifically affected by irradiation and/or infection, leading to a clearer separation of physiological profiles between modalities, as well as higher overall mortality, indicating increased vulnerability to experimental stress. This divergence cannot be attributed to methodological differences, as both experiments were conducted under strictly standardized conditions: controlled temperature, identical feeding protocols, and the use of bees of similar age. It therefore more likely reflects biological variability between the bee batches used in each experiment. A key factor lies in the temporal difference in bee collection: bees from Experiment A were collected in April, whereas those from Experiment B were collected in May. Although the experimental conditions were identical, this difference in collection date may have introduced seasonal effects on the bees’ baseline physiological state, related to photoperiod, foraging activity, or colony dynamics. Such seasonal variations could have influenced viral susceptibility or immune activity even before the onset of experimental treatments [38,39]. Other colony-specific characteristics may also help explain the observed variability. Colonies may differ in their prior exposure to anthropogenic stressors, such as pesticides, which can modulate physiological resilience [40]. Similarly, variations in natural pathogen load, particularly *Varroa destructor*, can alter the bees’ immune status before exposure to experimental stressors [41]. Genetic diversity represents another potentially important factor. Queens mate with multiple males, producing patrilines with varying sensitivities to stress. Patriline composition may fluctuate over time between colonies, thereby influencing the overall physiological response [42]. Taken together, these elements suggest that the stronger effects observed in Experiment B, reflect true biological variability between bee batches. However, they also highlight the challenge of reproducibility inherent to ecotoxicological studies involving live organisms. These results emphasize that even under strictly controlled experimental conditions, physiological responses may differ between trials due to natural biological complexity.

Overall, certain physiological responses in infected and/or irradiated bees reveal similar responses. In the midgut, GP and GR activities were stable (exp. A), indicating that reduced glutathione regeneration is not significantly impaired. This stability could reflect a local compensatory mechanism that maintains redox homeostasis despite exposure to stress. In the head (exp. A), no significant difference in G6PDH was found between the modalities, suggesting that neuronal antioxidant capacities are preserved, possibly through specific tissue protection mechanisms. Conversely, a decrease in G6PDH activity was observed in the abdomen (exp. B), a pattern also described in bees exposed to various pesticides [14,43]. This reduction could reflect a metabolic readjustment in favor of glycolysis, favoring rapid ATP production at the expense of the pentose phosphate pathway and thus the generation of NADPH, which is essential for maintaining glutathione-dependent antioxidant defenses. The kinetics of G6PDH also revealed a progressive decrease over time, suggesting that a persistent stress could reinforce this glycolytic orientation. At the metabolic level, the absence of significant differences in TG (abdomen), LDH (head), and GaPDH (abdomen) among modalities indicated that energetic demands were likely met without major metabolic rearrangements, with active glycolysis probably sufficient to sustain energy production [44]. The concomitant decrease in G6PDH further supports this hypothesis of glycolytic prioritization under chronic stress conditions. Regarding immune and detoxification markers, POX activity remained stable at day 14 (exp. A), consistent with previous findings reporting non-significant effects following infections or combined exposures to pesticides [14,45]. In contrast, CaE1 activity was reduced in the abdomen. Given the role of carboxylesterases in detoxification, metabolism, and immune signaling processes, this decrease could result from cellular exhaustion or a metabolic resource reallocation toward maintaining vital functions. Other biomarkers, discussed in the following sections, nevertheless displayed differentiated responses, reflecting specific regulatory mechanisms depending on the nature of the stress.

Infection by *Vairimorpha ceranae* resulted in a progressive increase in spore load, confirming active parasite proliferation [46,47], as well as an increase in mortality, consistent with previous findings [5,12,48]. However, the effects on syrup consumption differed between experiments: a decrease in Experiment A but hyperphagia in Experiment B, as previously reported in some infection cases [6,12], while a meta-analysis did not reveal any systematic effect [49]. These variations may have reflected genetic differences among colonies, as shown by Fontbonne *et al.* [41]. In the head, infection led to a moderate activation of antioxidant defenses. The activities of CAT and GST did not differ significantly between infected and control bees, consistent with observations in drones by Kairo *et al.* [45]. However, an early increase in CAT over time, as well as a higher SOD activity compared to controls were detected, suggesting a rapid antioxidant response to the rise in oxidative stress induced by the parasite [43,45]. These results suggested a protective neuronal activation aimed at preventing oxidative damage in the brain. The absence of significant effects on AChE, consistent with Kairo *et al.* [45] and Almasri *et al.* [14], further confirmed that cholinergic activity and neuronal function were globally preserved despite infection. In the abdomen, the infection appeared to reflect a gradual energetic constraint rather than a metabolic collapse. The progressive decrease in GaPDH and ATP over time suggested a slowdown in glycolytic flux and a reduction in energy capacity. This decline may have indicated that infected bees struggled to maintain metabolic balance under chronic stress. Such energetic limitation could have restricted the mobilization of immune defenses, as suggested by the decrease in POx, indicative of immunosuppression previously reported under *V. ceranae* infection [5,50]. Meanwhile, intestinal ALP tended to increase, indicating a potential immune activation [51] or a supportive mechanism to maintain cellular homeostasis [52]. CaE3 showed a marked decrease in both abdomen and intestine, in line with Dussaubat *et al.* [53], who reported a similar effect in infected queens. These results may have indicated an overall weakening of detoxification capacities. This reduction could have resulted from oxidative inactivation or from a shortage of energetic substrates necessary for enzymatic synthesis, reflecting a progressive depletion of metabolic resources and a loss of efficiency in enzymatic defense systems under chronic infection.

Chronic gamma irradiation, at both tested dose rates (14 µGy/h and 14 × 10³ µGy/h), neither significantly reduced nor stimulated parasite development. This result was expected, as the cumulative doses applied (0.005–4.7 Gy) were far below the acute doses used for pathogen sterilization in the food industry [54]. For instance, irradiation at 25 kGy completely inactivates *V. ceranae* spores [55]. Although the doses tested here were too low to directly affect the parasite, chronic sublethal exposure could subtly influence spore viability or virulence, an hypothesis that warrants further investigation. Among the two experiments conducted at 14 × 10³ µGy/h, only Experiment B showed an increase in mortality and changes in syrup consumption, suggesting initial sensitivity differences depending on the collection period. At the physiological level, in the head, the absence of significant effects on AChE suggested preserved neural functions, as observed in rats [56], zebrafish [57], and honeybees [19]. Similarly, the antioxidant enzymes CAT, SOD, and GST did not differ from controls after 14 days. This stability might reflect an early transient antioxidant response, as reported by Gagnaire et al. [19], who found significant effects on CAT on day 10 and on GST on day 3, but not after 14 days of irradiation. In parallel, the slight decrease in GaPDH, associated with higher ATP levels compared to infected bees, may indicate that irradiation partially limited glycolysis while maintaining cellular energy through compensatory mitochondrial mechanisms or reduced energy expenditure. In the abdomen, the decrease in G6PDH suggested reduced NADPH production, which is essential for glutathione regeneration and neutralization of reactive oxygen species (ROS). This redox deficit could have inhibited GaPDH and contributed to the slowdown of glycolytic flux, reflecting a state of chronic energetic constraint [58–60]. Meanwhile, the decrease in POX activity suggested an immunosuppressive effect, as observed in *Spodoptera littoralis* [61] and *Apis mellifera* [19]. One possible hypothesis is that, in the absence of direct immune stimulation such as pathogen infection, the organism may voluntarily downregulate POX activity to conserve energetic resources and redirect them toward essential processes like cellular repair or oxidative stress regulation. Such adaptive energy reallocation has already been reported in certain insects, including *Forficula auricularia* during reproduction [62]. In the midgut, ALP tended to increase, particularly at low dose rates, which could reflect local immune activation or a reparative response [51,52]. However, this adaptation appeared limited by a decrease in CaE1 and CaE3 activities, indicating reduced detoxification capacity. At high dose rates, only CaE3 decreased in the midgut, consistent with the results of Gagnaire et al. [19], whereas at low dose rates, both CaE1 and CaE3 were lower, suggesting a greater sensitivity of digestive tissues to prolonged exposure.

Under combined stress, no significant interaction between infection and irradiation was observed for spore load or mortality. In contrast, an antagonistic interaction was detected for syrup consumption: bees simultaneously exposed to infection and irradiation consumed less than those subjected to a single stress. This reduction could reflect a disruption of energy metabolism, possibly linked to damage in digestive tissues or hormonal dysregulation of satiety (vitellogenin, ILPs) [63]. An antagonistic interaction between infection and irradiation was also observed for SOD and GST (head) and G6PDH (abdomen), suggesting oxidative stress exceeding regulatory capacity and leading to saturation of antioxidant defenses. Conversely, the higher CAT activity observed in infected and irradiated bees may reflect a compensatory activation of the catalase pathway aimed at maintaining global redox balance. Elevated AChE activity under combined stress could similarly reflect an indirect response to oxidative stress [64] or activation of apoptotic pathways [65], potentially altering neurotransmission and the central regulation of metabolic and immune functions [66,67]. In the abdomen, ATP levels were significantly higher under combined stress, indicating a synergistic interaction between infection and irradiation. This increase may reflect sustained energetic compensation through mitochondrial adaptation, allowing the maintenance of sufficient energy supply despite metabolic disruptions induced by dual stress [68]. At the immune level, antagonistic interactions between infection and irradiation were observed for POX and ALP (exp. B). POX exhibited an early increase followed by a progressive decrease, reflecting an initially amplified activation followed by exhaustion or adaptive regulation to limit the energetic cost of a prolonged response. ALP showed the opposite dynamic, with an initial decrease followed by a gradual recovery, indicating local reorganization of immune activity. This contrasted kinetics is consistent with the concept of immunological plasticity in insects, in which immune responses are modulated according to the intensity, duration, and nature of environmental and metabolic constraints [69]. CaE1 and CaE3 showed several antagonistic interactions depending on the dose rate. At high dose rates, CaE3 decreased in both the intestine and abdomen (exp. A), whereas CaE1 and CaE3 were reduced in the abdomen (exp. B). At low dose rates, CaE1 also decreased in the intestine and abdomen (exp. A). These results may indicate a coordinated alteration of detoxification capacities, probably linked to metabolic saturation or oxidative inactivation, marking the limit of adaptive mechanisms under combined stress.

The results of multivariate analyses on biomarkers indicate that exposure to a single stressor already modifies the biomarkers profile of bees, although in Experiment A, these variations remain weak between conditions. In Experiment B, however, the impact of each stress is reflected by a more distinct separation, particularly between the control and the combined stress condition. Biologically, observations suggest that infection and irradiation have distinct impacts. Indeed, infection with *Vairimorpha ceranae* is known to alter the metabolism and immune defenses of bees [5,50], which can lead to changes in the activity of biomarkers related to the oxidative stress response. Prolonged irradiation can generate chronic stress, potentially inducing compensatory mechanisms that may, in turn, lead to unexpected variations in biomarker activities. A more substantial divergence from controls was observed in bees subjected to both infection and irradiation, suggesting that combined stress may induce broader physiological alterations. Although such a pattern was not evident when analyzing individual biomarkers separately, a multibiomarker approach appears more effective in capturing these cumulative variations. This indicates that immunity, metabolic processes, and antioxidant responses may be more extensively modulated under combined stress conditions. From an ecological and health perspective, the co-occurrence of stressors such as *V. ceranae* and chronic irradiation could amplify the deleterious effects on bees, with potential cascading effects on pollination services and ecosystem resilience.

## Conclusion

The combination of *Vairimorpha ceranae* infection and chronic gamma irradiation in *Apis mellifera* induces complex, predominantly antagonistic physiological disturbances, distinct from those caused by each stressor individually. These interactions, which strongly depend on the bees’ initial physiological state, reflect a systemic disruption of metabolic, antioxidant, immune, and detoxification functions, going beyond a simple additive effect of individual stressors. These findings highlight the need to reassess conventional risk assessment approaches, which are often focused on isolated exposures, by integrating combined effects, nonlinear interactions, and natural biological variability into ecotoxicological protocols. They also pave the way for further research to better understand how multiple environmental stressors shape the physiological and ecological resilience of pollinators.

## Supporting information

S1 Text Automated spore counting method using deep learning. This supplementary text details the deep learning-based approach used to automatically count *Vairimorpha* spores from high-resolution microscopic images.
(PDF)

S1 Fig Effects of ionizing radiation and *Vairimorpha ceranae* infection on biomakers. One day after emergence, the bees were infected with *V. ceranae* and, three days after infection, were continuously exposed to gamma radiation for 14 days. C: Control bees, neither irradiated nor infected. V: Bees only infected. L: Bees only irradiated at 14 µGy/h. VL: Bees both infected and irradiated at 14 µGy/h. H: Bees only irradiated at 14 × 10³ µGy/h. VH: Bees both infected and irradiated at 14 × 10³ µGy/h. Midgut GR activity (A), Midgut GP activity (B), Head G6PDH activity (C), Head LDH activity (D), Abdomen TG content (E), Abdomen CaE3 activity (F) and Abdomen Pox activity (G) were measured after 14 days of irradiation in experiment A. Data represent the mean activity from 6 tissues extracts tested in triplicate ± SD per cage (4 cages/modality).
(PDF)

S1 Table Significant effects of irradiation and/or infection on the parameters tested in Experiment A. C: Control bees, neither irradiated nor infected. V: Bees only infected. L: Bees only irradiated at 14 µGy/h. VL: Bees both infected and irradiated at 14 µGy/h. H: Bees only irradiated at 14 mGy/h. VH: Bees both infected and irradiated at 14 mGy/h. Results show significant effects for all factors tested, with significance levels indicated as follows: (*): p < 0.05; (**): p < 0.01; (***): p < 0.001; NS: not significant.
(PDF)

S2 Table Significant effects of irradiation and/or infection on the parameters tested in Experiment B. C: Control bees, neither irradiated nor infected. V: Bees only infected. H: Bees only irradiated at 14 mGy/h. VH: Bees both infected and irradiated at 14 mGy/h. NS: not available. Results show significant effects for all factors tested, with significance levels indicated as follows: (*): p < 0.05; (**): p < 0.01; (***): p < 0.001; NS: not significant.
(PDF)

S3 Table Raw data of syrup consumption and mortality from Experiment A. C: Control bees, neither irradiated nor infected. V: Bees only infected. L: Bees only irradiated at 14 µGy/h. VL: Bees both infected and irradiated at 14 µGy/h. H: Bees only irradiated at 14 mGy/h. VH: Bees both infected and irradiated at 14 mGy/h. NA: not available.
(PDF)

S4 Table Raw data of spore numbers from Experiment A. C: Control bees, neither irradiated nor infected. V: Bees only infected. L: Bees only irradiated at 14 µGy/h. VL: Bees both infected and irradiated at 14 µGy/h. H: Bees only irradiated at 14 mGy/h. VH: Bees both infected and irradiated at 14 mGy/h. NA: not available.
(PDF)

S5 Table Raw data of biomarkers from Experiment A. C: Control bees, neither irradiated nor infected. V: Bees only infected. L: Bees only irradiated at 14 µGy/h. VL: Bees both infected and irradiated at 14 µGy/h. H: Bees only irradiated at 14 mGy/h. VH: Bees both infected and irradiated at 14 mGy/h. NA: not available.
(PDF)

S6 Table Raw data of syrup consumption and mortality from Experiment B. C: Control bees, neither irradiated nor infected. V: Bees only infected. H: Bees only irradiated at 14 mGy/h. VH: Bees both infected and irradiated at 14 mGy/h. NA: not available.
(PDF)

S7 Table Raw data of spore numbers from Experiment B. C: Control bees, neither irradiated nor infected. V: Bees only infected. H: Bees only irradiated at 14 mGy/h. VH: Bees both infected and irradiated at 14 mGy/h. NA: not available.
(PDF)

S8 Table Raw data of biomarkers from Experiment B. C: Control bees, neither irradiated nor infected. V: Bees only infected. H: Bees only irradiated at 14 mGy/h. VH: Bees both infected and irradiated at 14 mGy/h. NA: not available.
(PDF)
