# Supplementary material for: Interactions between ionizing radiation and Vairimorpha (Nosema) ceranae on the honeybee, Apis mellifera L
Source: PLoS One. 2026 Jan 9;21(1):e0339853. doi: 10.1371/journal.pone.0339853 (PMC12788649; doi:10.1371/journal.pone.0339853)
Supplement: S1 Text — This supplementary text details the deep learning-based approach used to automatically count Vairimorpha spores from high-resolution microscopic images. (PDF) [file pone.0339853.s001.pdf]

S1 Text. Automated Spore Counting Method Using Deep Learning. This supplementary text details the deep learning-based approach used to automatically count *Vairimorpha* spores from high-resolution microscopic images.

Photos were taken and analysed using AI to count the number of spores per image. High-resolution images (4648×3506 pixels) were captured and manually annotated to create a ground truth dataset. To manage the large image sizes, a tiling approach was used, dividing each image into 256×256 pixel patches. These patches were then used to train a deep learning model based on a pre-trained U-Net architecture [1], with an EfficientNetB5 [2] encoder backbone sourced from the Segmentation Models library [3]. The model was trained using the Adam optimizer, with a combined Binary Cross-Entropy and Jaccard loss function. Data augmentation techniques included symmetry transformations, rotation, random brightness and contrast adjustments, as well as random noise. The training process used a learning rate of $1x10^{-4}$ and a batch size of 32 images, running on an NVIDIA RTX A6000 GPU. During inference, Test Time Augmentation (TTA) [4] was applied using eight combinations of rotation and symmetry, and predictions were retained if a spore was detected in at least three out of the eight TTA outputs. On the test set, the model achieved strong performance metrics, with an F1 score of 0.962, an accuracy of 0.969, a precision of 0.955, and a recall of 0.969.

References

[1] Ronneberger O, Fischer P, Brox T. U-Net: Convolutional Networks for Biomedical Image Segmentation 2015. https://doi.org/10.48550/arXiv.1505.04597.
[2] Tan M, Le QV. EfficientNet: Rethinking Model Scaling for Convolutional Neural Networks 2020. https://doi.org/10.48550/arXiv.1905.11946.
[3] Iakubovskii P. qubvel/segmentation_models 2025.
[4] Kimura M. Understanding Test-Time Augmentation 2024. https://doi.org/10.48550/arXiv.2402.06892.
