# Supplementary material for: Interactions between ionizing radiation and Vairimorpha (Nosema) ceranae on the honeybee, Apis mellifera L
Source: PLoS One. 2026 Jan 9;21(1):e0339853. doi: 10.1371/journal.pone.0339853 (PMC12788649; doi:10.1371/journal.pone.0339853)
Supplement: S1 Fig — One day after emergence, the bees were infected with V. ceranae and, three days after infection, were continuously exposed to gamma radiation for 14 days. C: Control bees, neither irradiated nor infected. V: Bees only infected. L: Bees only irradiated at 14 µGy/h. VL: Bees both infected and irradiated at 14 µGy/h. H: Bees only irradiated at 14 × 10³ µGy/h. VH: Bees both infected and irradiated at 14 × 10³ µGy/h. Midgut GR activity (A), Midgut GP activity (B), Head G6PDH activity (C), Head LDH activity (D), Abdomen TG content (E), Abdomen CaE3 activity (F) and Abdomen Pox activity (G) were measured after 14 days of irradiation in experiment A. Data represent the mean activity from 6 tissues extracts tested in triplicate ± SD per cage (4 cages/modality). (PDF) [file pone.0339853.s002.pdf]

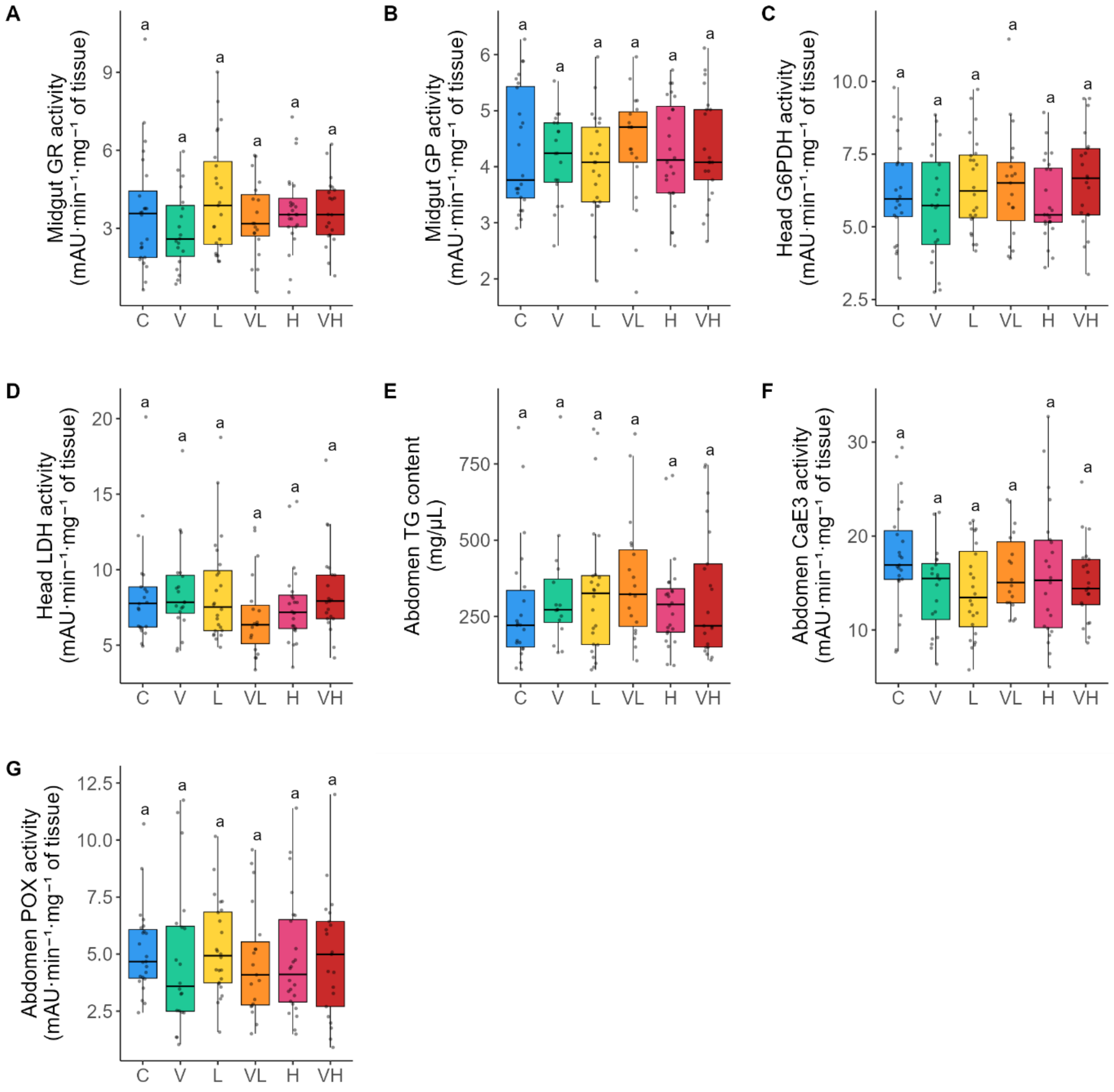

S1 Fig. Effects of ionizing radiation and *Vairimorpha ceranae* infection on biomakers. One day after emergence, the bees were infected with *V. ceranae* and, three days after infection, were continuously exposed to gamma radiation for 14 days. C: Control bees, neither irradiated nor infected. V: Bees only infected. L: Bees only irradiated at 14 µGy/h. VL: Bees both infected and irradiated at 14 µGy/h. H: Bees only irradiated at 14 × 10^3 µGy/h. VH: Bees both infected and irradiated at 14 × 10^3 µGy/h. Midgut GR activity (A), Midgut GP activity (B), Head G6PDH activity (C), Head LDH activity (D), Abdomen TG content (E), Abdomen CaE3 activity (F) and Abdomen Pox activity (G) were measured after 14 days of irradiation in Experiment A. Data represent the mean activity from 6 tissues extracts tested in triplicate ± SD per cage (4 cages/modality).
