# Supplementary material for: Interactions between ionizing radiation and Vairimorpha (Nosema) ceranae on the honeybee, Apis mellifera L
Source: PLoS One. 2026 Jan 9;21(1):e0339853. doi: 10.1371/journal.pone.0339853 (PMC12788649; doi:10.1371/journal.pone.0339853)
Supplement: S1 Table — C: Control bees, neither irradiated nor infected. V: Bees only infected. L: Bees only irradiated at 14 µGy/h. VL: Bees both infected and irradiated at 14 µGy/h. H: Bees only irradiated at 14 mGy/h. VH: Bees both infected and irradiated at 14 mGy/h. Results show significant effects for all factors tested, with significance levels indicated as follows: (*): p < 0.05; (**): p < 0.01; (***): p < 0.001; NS: not significant. (PDF) [file pone.0339853.s003.pdf]

S1 Table. Significant effects of irradiation and/or infection on the parameters tested in Experiment A. C: Control bees, neither irradiated nor infected. V: Bees only infected. L: Bees only irradiated at 14 µGy/h. VL: Bees both infected and irradiated at 14 µGy/h. H: Bees only irradiated at 14 mGy/h. VH: Bees both infected and irradiated at 14 mGy/h. Results show significant effects for all factors tested, with significance levels indicated as follows: (*): $p < 0.05$; (**): $p < 0.01$; (***): $p < 0.001$; NS: not significant.

	C vs L	C vs H	C vs V	C vs VL	C vs VH	L vs H	L vs V	L vs VL	L vs VH	H vs V	H vs VL	H vs VH	V vs VL	V vs VH	VL vs VH
Spore numeration	NS	NS	***	***	***	NS	***	***	***	***	***	***	NS	NS	NS
Mortality	NS	NS	*	*	NS	NS	*	*	NS	*	*	NS	NS	NS	NS
Consumption	NS	NS	**	*	NS	NS	**	*	NS	***	**	*	NS	NS	NS
Abdomen POx	NS	NS	NS	NS	NS	NS	NS	NS	NS	NS	NS	NS	NS	NS	NS
Abdomen CaE1	NS	NS	NS	NS	NS	NS	NS	**	NS	NS	*	NS	NS	NS	NS
Abdomen CaE3	NS	NS	NS	NS	NS	NS	NS	NS	NS	NS	NS	NS	NS	NS	NS
Abdomen TG	NS	NS	NS	NS	NS	NS	NS	NS	NS	NS	NS	NS	NS	NS	NS
Midgut ALP	***	NS	NS	NS	NS	**	***	*	NS	NS	NS	NS	NS	NS	NS
Midgut GP	NS	NS	NS	NS	NS	NS	NS	NS	NS	NS	NS	NS	NS	NS	NS
Midgut GR	NS	NS	NS	NS	NS	NS	NS	NS	NS	NS	NS	NS	NS	NS	NS
Midgut CaE1	**	NS	NS	**	NS	NS	NS	NS	**	NS	NS	NS	NS	NS	***
Midgut CaE3	*	**	*	***	NS	NS	NS	NS	NS	NS	NS	NS	NS	NS	**
Head GaPDH	NS	NS	NS	NS	NS	NS	NS	NS	NS	*	NS	NS	NS	NS	NS
Head G6PDH	NS	NS	NS	NS	NS	NS	NS	NS	NS	NS	NS	NS	NS	NS	NS
Head ATP	NS	NS	NS	NS	NS	NS	*	NS	NS	**	NS	NS	NS	NS	NS
Head LDH	NS	NS	NS	NS	NS	NS	NS	NS	NS	NS	NS	NS	NS	NS	NS
