# Supplementary material for: Interactions between ionizing radiation and Vairimorpha (Nosema) ceranae on the honeybee, Apis mellifera L
Source: PLoS One. 2026 Jan 9;21(1):e0339853. doi: 10.1371/journal.pone.0339853 (PMC12788649; doi:10.1371/journal.pone.0339853)
Supplement: S2 Table — C: Control bees, neither irradiated nor infected. V: Bees only infected. H: Bees only irradiated at 14 mGy/h. VH: Bees both infected and irradiated at 14 mGy/h. NS: not available. Results show significant effects for all factors tested, with significance levels indicated as follows: (*): p < 0.05; (**): p < 0.01; (***): p < 0.001; NS: not significant. (PDF) [file pone.0339853.s004.pdf]

S2 Table. Significant effects of irradiation and/or infection on the parameters tested in Experiment B. C: Control bees, neither irradiated nor infected. V: Bees only infected. H: Bees only irradiated at 14 mGy/h. VH: Bees both infected and irradiated at 14 mGy/h. NS: not available. Results show significant effects for all factors tested, with significance levels indicated as follows: (*): $p < 0.05$; (**): $p < 0.01$; (***): $p < 0.001$; NS: not significant.

	C vs H	C vs V	C vs VH	H vs V	H vs VH	V vs VH
Spore numeration	NS	***	***	***	***	NS
Mortality	*	*	**	NS	NS	NS
Consumption	*	**	NS	NS	**	*
Head SOD	NS	*	NS	NS	NS	**
Head CAT	NS	NS	**	*	*	NS
Head GST	NS	NS	NS	NS	**	NS
Abdomen G6PDH	***	***	***	*	***	NS
Abdomen GaPDH	NS	NS	NS	**	NS	NS
Abdomen ATP	NS	NS	***	NS	***	***
Abdomen POx	***	***	NS	NS	**	*
Midgut ALP	**	*	NS	NS	*	NS
Head AChE	NS	NS	*	NS	NS	*
Abdomen CaE1	***	***	***	*	NS	NS
Abdomen CaE3	NS	***	NS	***	NS	***
