# Supplementary material for: Interactions between ionizing radiation and Vairimorpha (Nosema) ceranae on the honeybee, Apis mellifera L
Source: PLoS One. 2026 Jan 9;21(1):e0339853. doi: 10.1371/journal.pone.0339853 (PMC12788649; doi:10.1371/journal.pone.0339853)
Supplement: S3 Table — C: Control bees, neither irradiated nor infected. V: Bees only infected. L: Bees only irradiated at 14 µGy/h. VL: Bees both infected and irradiated at 14 µGy/h. H: Bees only irradiated at 14 mGy/h. VH: Bees both infected and irradiated at 14 mGy/h. NA: not available. (PDF) [file pone.0339853.s005.pdf]

S3 Table. Raw data of syrup consumption and mortality from Experiment A. C: Control bees, neither irradiated nor infected. V: Bees only infected. L: Bees only irradiated at 14 µGy/h. VL: Bees both infected and irradiated at 14 µGy/h. H: Bees only irradiated at 14 mGy/h. VH: Bees both infected and irradiated at 14 mGy/h. NA: not available.

Modality	Days of irradiation	Sirup consumption (µL/bee)	Number of dead bees	Number of live bees
C	0	38.5	0	48
C	0	47.9	0	49
C	0	41.7	0	49
C	0	68.4	0	42
V	0	51.3	0	52
V	0	50.1	0	53
V	0	44.3	0	63
V	0	48.8	0	48
L	0	43.9	0	49
L	0	49.7	0	46
L	0	41.6	0	57
L	0	41.4	0	52
VL	0	50.9	0	54
VL	0	57.4	0	52
VL	0	45.8	0	48
VL	0	48.2	0	50
H	0	50.2	0	43
H	0	45.5	0	51
H	0	48.5	0	53
H	0	49	0	50
VH	0	62.4	0	49
VH	0	58.1	0	56
VH	0	57.4	0	50
VH	0	44.5	0	52
C	2	75.5	0	48
C	2	70.8	0	49
C	2	81.4	0	49
C	2	76.8	0	42
V	2	81	0	52
V	2	68.2	0	53
V	2	60.5	0	63
V	2	82.1	0	48
L	2	84.5	0	49
L	2	74.5	0	46
L	2	85	0	57
L	2	76.5	0	52

VL	2	90.5	0	54
VL	2	87.8	0	52
VL	2	67.6	0	48
VL	2	72.5	0	50
H	2	74.2	0	43
H	2	80	0	51
H	2	83.8	0	53
H	2	78.9	0	50
VH	2	77.7	0	49
VH	2	71.2	0	56
VH	2	87	0	50
VH	2	89.4	0	52
C	4	106.2	0	48
C	4	108.6	0	49
C	4	103.2	0	49
C	4	121	0	42
V	4	84.6	1	51
V	4	87.9	0	53
V	4	96.7	2	61
V	4	99.2	2	46
L	4	126.1	0	49
L	4	114.4	0	46
L	4	86.1	1	56
L	4	111.7	0	52
VL	4	88.3	2	52
VL	4	89.7	0	52
VL	4	98.9	0	48
VL	4	104.3	4	46
H	4	112.9	1	42
H	4	135.7	0	51
H	4	90.7	0	53
H	4	110.3	0	50
VH	4	98.2	0	49
VH	4	87.8	2	54
VH	4	93.4	0	50
VH	4	100.8	0	52
C	7	127.9	4	44
C	7	100.3	2	47
C	7	104.5	5	44
C	7	177.4	1	41
V	7	84.5	7	45
V	7	78.2	5	48
V	7	75.7	6	57

V	7	103.5	6	42
L	7	75.1	0	49
L	7	144.4	0	46
L	7	89.5	2	55
L	7	154	5	47
VL	7	86	10	44
VL	7	82.9	8	44
VL	7	104.9	2	46
VL	7	79	12	38
H	7	103.1	2	41
H	7	117.2	1	50
H	7	113.8	0	53
H	7	151.3	7	43
VH	7	83.5	1	48
VH	7	94.6	5	51
VH	7	106.7	8	42
VH	7	120.1	6	46
C	9	92.8	10	38
C	9	65.3	2	47
C	9	99.1	7	42
C	9	98.3	2	40
V	9	84.1	13	39
V	9	68.8	13	40
V	9	79.8	13	50
V	9	74.5	17	31
L	9	84.6	2	47
L	9	102.4	2	44
L	9	77.9	6	51
L	9	93	9	43
VL	9	43.5	17	37
VL	9	56.6	16	36
VL	9	79.1	6	42
VL	9	66.8	18	32
H	9	79.8	2	41
H	9	114.2	4	47
H	9	82.3	4	49
H	9	89.4	8	42
VH	9	67.3	12	37
VH	9	69.4	11	45
VH	9	86.6	11	39
VH	9	65.1	11	41
C	11	95.1	12	36
C	11	68.5	6	43

C	11	93.7	9	40
C	11	96.9	7	35
V	11	91.9	17	35
V	11	91.9	21	32
V	11	98.7	22	41
V	11	98.4	24	24
L	11	101.9	4	45
L	11	91.9	4	42
L	11	67	10	47
L	11	99.7	9	43
VL	11	56.8	22	32
VL	11	87.1	25	27
VL	11	102.8	9	39
VL	11	112.6	22	28
H	11	95.7	5	38
H	11	112.1	6	45
H	11	85.1	5	48
H	11	94.4	9	41
VH	11	84.1	16	33
VH	11	79	17	39
VH	11	114.8	14	36
VH	11	67.3	17	35
C	14	83.3	16	32
C	14	136.8	8	41
C	14	161.5	11	38
C	14	134	8	34
V	14	111.7	25	27
V	14	77.8	31	22
V	14	116.1	36	27
V	14	114.5	26	22
L	14	141.9	6	43
L	14	130.7	5	41
L	14	144.6	15	42
L	14	154.4	16	36
VL	14	104	27	27
VL	14	120.8	34	18
VL	14	142.6	15	33
VL	14	150.4	29	21
H	14	165.3	6	37
H	14	161.6	8	43
H	14	126.5	13	40
H	14	140.4	11	39
VH	14	103.2	27	22

VH	14	126.1	32	24
VH	14	139.4	22	28
VH	14	99.3	25	27
