# Supplementary material for: Interactions between ionizing radiation and Vairimorpha (Nosema) ceranae on the honeybee, Apis mellifera L
Source: PLoS One. 2026 Jan 9;21(1):e0339853. doi: 10.1371/journal.pone.0339853 (PMC12788649; doi:10.1371/journal.pone.0339853)
Supplement: S4 Table — C: Control bees, neither irradiated nor infected. V: Bees only infected. L: Bees only irradiated at 14 µGy/h. VL: Bees both infected and irradiated at 14 µGy/h. H: Bees only irradiated at 14 mGy/h. VH: Bees both infected and irradiated at 14 mGy/h. NA: not available. (PDF) [file pone.0339853.s006.pdf]

S4 Table. Raw data of spore numbers from Experiment A. C: Control bees, neither irradiated nor infected. V: Bees only infected. L: Bees only irradiated at 14 µGy/h. VL: Bees both infected and irradiated at 14 µGy/h. H: Bees only irradiated at 14 mGy/h. VH: Bees both infected and irradiated at 14 mGy/h. NA: not available.

Modality	Days of irradiation	Spore numbers (x10^6 spores/bee)
C	14	1.1
C	14	1
C	14	1.8
C	14	2
C	14	1.6
C	14	0.8
C	14	1.5
C	14	1.7
C	14	2
C	14	1.7
C	14	0.8
C	14	0.9
C	14	2
C	14	1.4
C	14	1
C	14	1.3
C	14	1.4
C	14	1.4
C	14	1.7
C	14	1.6
V	14	43.5
V	14	49.5
V	14	47.4
V	14	31.2
V	14	41.9
V	14	28.2
V	14	35.8
V	14	32.2
V	14	34.9
V	14	38.5
V	14	22.1
V	14	23.1
V	14	22.1
V	14	22.4
V	14	25.8
V	14	28.3

V	14	28.4
V	14	30.8
V	14	29.5
V	14	26.7
L	14	1.2
L	14	1.6
L	14	1.7
L	14	1.6
L	14	1.3
L	14	1.7
L	14	2
L	14	2.8
L	14	2.6
L	14	2.6
L	14	0.8
L	14	1.5
L	14	1.6
L	14	1.7
L	14	1.9
L	14	0.7
L	14	1.4
L	14	1.9
L	14	2
L	14	2.1
VL	14	27.9
VL	14	29.2
VL	14	33.5
VL	14	30.9
VL	14	28.3
VL	14	22.4
VL	14	24.8
VL	14	27
VL	14	23.3
VL	14	19.7
VL	14	35.7
VL	14	46.2
VL	14	42.4
VL	14	43.9
VL	14	41.6
VL	14	18.6
VL	14	23.9
VL	14	25.4
VL	14	26.7

VL	14	27.2
H	14	0.8
H	14	1.3
H	14	1.4
H	14	1.2
H	14	1.5
H	14	0.6
H	14	1.1
H	14	1.4
H	14	1.3
H	14	1.2
H	14	1.8
H	14	2
H	14	2.1
H	14	2.7
H	14	2.4
H	14	0.9
H	14	0.7
H	14	0.6
H	14	0.9
H	14	0.9
VH	14	31.5
VH	14	36
VH	14	35.7
VH	14	40.4
VH	14	36.9
VH	14	39.3
VH	14	46.5
VH	14	48.9
VH	14	49.3
VH	14	51.7
VH	14	29.9
VH	14	35.2
VH	14	35
VH	14	37.6
VH	14	37.7
VH	14	34.2
VH	14	34.2
VH	14	29.4
VH	14	35.5
VH	14	34.3
