# Supplementary material for: Interactions between ionizing radiation and Vairimorpha (Nosema) ceranae on the honeybee, Apis mellifera L
Source: PLoS One. 2026 Jan 9;21(1):e0339853. doi: 10.1371/journal.pone.0339853 (PMC12788649; doi:10.1371/journal.pone.0339853)
Supplement: S5 Table — C: Control bees, neither irradiated nor infected. V: Bees only infected. L: Bees only irradiated at 14 µGy/h. VL: Bees both infected and irradiated at 14 µGy/h. H: Bees only irradiated at 14 mGy/h. VH: Bees both infected and irradiated at 14 mGy/h. NA: not available. (PDF) [file pone.0339853.s007.pdf]

S5 Table. Raw data of biomarkers from Experiment A. C: Control bees, neither irradiated nor infected. V: Bees only infected. L: Bees only irradiated at 14 µGy/h. VL: Bees both infected and irradiated at 14 µGy/h. H: Bees only irradiated at 14 mGy/h. VH: Bees both infected and irradiated at 14 mGy/h. NA: not available.

Modality	Days of irradiation	Abdomen Pox activity (mAU. $min^{-1}.mg^{-1}$ of tissue)	Abdomen CaE1 activity (mAU.mg^{-1} of tissue)	Abdomen CaE3 activity (mAU. $min^{-1}.mg^{-1}$ of tissue)	Abdomen TG level (mAU.mg^{-1} of tissue)	Midgut ALP activity (mAU. $min^{-1}.mg^{-1}$ of tissue	Midgut GP activity (mAU. $min^{-1}.mg^{-1}$ of tissue	Midgut GP activity (mAU. $min^{-1}.mg^{-1}$ of tissue	Midgut CaE1 activity (mAU.mg^{-1} of tissue)	Midgut CaE3 activity (mAU. $min^{-1}.mg^{-1}$ of tissue	Head GaPDH activity (mAU. $min^{-1}.mg^{-1}$ of tissue	Head G6PDH activity (mAU. $min^{-1}.mg^{-1}$ of tissue	Head ATP activity (LI.mg^{-1} of tissue)	Head LDH activity (mAU. $min^{-1}.mg^{-1}$ of tissue
C	14	4.9	0.5	15.2	227.4	11.2	3.4	6.4	1.1	43.5	147.5	6.4	56.3	9.7
C	14	4.9	0.5	15.2	227.4	11.2	3.4	6.4	1.1	43.5	147.5	6.4	56.3	9.7
C	14	3.9	0.6	20.2	869	10.8	3.6	5	1	41	138	4.3	61.1	6.1
C	14	6.2	0.6	29.4	76.3	10.7	2.9	1.9	1.2	58.9	141.2	5.9	63.5	6.2
C	14	4.9	0.5	15.2	227.4	11.2	3.4	6.4	1.1	43.5	147.5	6.4	56.3	9.7
C	14	3.9	0.6	20.2	869	10.8	3.6	5	1	41	138	4.3	61.1	6.1
C	14	6.2	0.6	29.4	76.3	10.7	2.9	1.9	1.2	58.9	141.2	5.9	63.5	6.2
C	14	4.5	0.6	22.9	524.8	12.6	3.7	2.3	1.1	44.4	125.5	4.1	59.7	6.2
C	14	3.8	0.6	16.4	164.7	11.9	5.9	6	1.1	43.3	NA	NA	NA	NA
C	14	5.9	0.8	25.6	254.1	9.8	5.6	3.8	1.1	37.5	283.1	8.7	242.9	7.4
C	14	6	0.6	15.4	145.6	12.6	6.3	10.3	1.3	47.3	NA	NA	NA	NA
C	14	4	0.6	17.9	128	10.4	5.4	0.6	1.1	46.9	276.9	7.1	159.7	5.1
C	14	NA	NA	NA	NA	10.1	3.6	4.2	1.1	44.5	160	8.3	78.8	20.1
C	14	4	0.5	7.7	221.2	5.1	3.1	3.6	1	30	185.9	6.3	81.2	12.2
C	14	5.4	0.6	21	301	7.5	3.5	2.6	1.2	38.8	102.8	6	81.3	8.2
C	14	4.2	0.6	15.8	347.3	10.8	3.1	5.7	1.2	46.7	156.1	5.8	90	13.6
C	14	2.4	0.6	16.9	235.8	8.9	5.65	1.9	1.3	55.9	231.4	7.1	97.1	6.3
C	14	8.8	0.6	16.5	439.1	11.4	4.9	1.7	1.3	53.1	240.8	5.3	96	7.8
C	14	NA	NA	NA	NA	11.8	4.4	1.5	1.2	65.1	314.5	5.5	84.3	7.8
C	14	10.7	0.7	28.5	392.7	12	5.9	2.2	1.2	46.1	243.1	9.8	78.5	NA

C	14	6.7	0.5	10.6	222.2	8.9	3.2	3.5	0.9	42.4	207.1	4.2	86.3	9.7
C	14	4.7	0.5	7.9	143.4	4	3.8	3.8	0.9	29.4	171.8	3.2	87.1	8.7
C	14	3	0.6	15.4	171.1	6.2	3.5	7.1	0.9	27.1	212.6	5.4	136	8.6
C	14	6.2	0.6	16.7	207.3	9	3.6	3.6	1	36.5	229	5.7	132.4	8.9
C	14	4.6	0.6	17.7	741.4	8.9	3.2	1.8	0.9	26.1	NA	NA	NA	NA
C	14	5.9	0.7	19.4	NA	8.2	5.5	3.8	1.2	52.4	290.2	7.2	137.6	5.8
C	14	2.8	0.6	18.3	164	4.5	4.8	2.4	1.1	40.6	294.1	7.7	134.5	6
C	14	3.5	0.5	11.5	81	NA	NA	NA	NA	NA	323.9	8.8	145.2	7.5
C	14	6.5	0.6	23.7	99.1	7.2	4.7	0.9	1	30.4	183.5	4.4	114.9	4.9
V	14	2.5	0.6	13.1	272.6	10.7	3.8	4	1.1	31.9	174.9	3.1	59.2	8.8
V	14	1.4	0.6	8.5	134.8	9.1	4.1	5	1	33.7	123.9	5.7	58.8	6.8
V	14	1	0.6	15.6	362	9.4	4.9	6	1	33.7	215.7	5.7	69.2	7.2
V	14	3.3	0.6	16.9	238	6.7	3.7	3.5	0.9	19.2	167.8	4.6	61.9	9.8
V	14	4.7	0.6	18.2	433.4	7.2	4.9	4.6	1	35.8	312.9	8.2	190	7.8
V	14	2.4	0.6	17.5	405.7	7.4	4.6	2.8	1.1	44.4	289.4	6.1	91.2	7.8
V	14	3.7	0.7	16	515.3	NA	NA	NA	NA	NA	93.3	6.7	87.3	4.8
V	14	3.3	0.5	8.1	153.5	9.3	4.8	2.6	1	39.2	276.1	7.5	84.8	7.5
V	14	1.4	0.5	9.2	287.2	8.3	4.5	3.8	1.1	29.2	137.3	4.9	77.1	7.6
V	14	4.6	0.5	11.7	288.2	8.8	3.8	2.4	1.1	36.9	158.4	3.8	73.6	8.9
V	14	6.1	0.5	9	904.3	NA	NA	NA	NA	NA	123.9	2.8	99.3	12.5
V	14	2.5	0.5	6.4	251.5	10.3	2.6	1	1.2	33.9	NA	NA	NA	NA
V	14	2.5	0.6	11.9	NA	8.9	4.6	2.3	1.1	46.9	280	6.3	78.8	6.7
V	14	6.3	0.6	22.5	NA	8	3.1	1.4	1.2	49.6	329.4	8.9	100.8	12.6
V	14	6.9	0.6	17.5	NA	NA	NA	NA	NA	NA	NA	NA	NA	NA
V	14	6.2	0.6	15.9	NA	11.3	4.9	0.9	1.2	34.1	NA	NA	NA	NA
V	14	11.2	0.6	16.5	270	6	3.3	5.3	0.9	22.2	201.6	2.8	78	5.2
V	14	3.5	0.7	22.3	272.2	10.8	4.2	2.1	1	33.1	176.5	4.5	107.7	9.6

V	14	NA	NA	NA	NA	NA	NA	NA	NA	NA	254.1	4.2	88.3	7.5
V	14	10.3	0.5	14.8	209.7	9.4	4.8	2.5	1.2	43.5	288.6	7.8	112.5	4.6
V	14	11.8	0.6	15.4	132	9.7	5.5	3.1	1.3	43.9	262	5.7	116	9.8
V	14	NA	NA	NA	NA	7.2	4.2	1.7	1.1	33.7	222.8	7.1	114.3	8.6
V	14	NA	NA	NA	NA	9.5	3.3	1.2	1.2	55.9	339.6	8.6	170	17.9
L	14	3.9	0.5	16.7	377.9	14.4	3.8	9	0.9	49.2	163.9	6.1	113.3	7.7
L	14	3.6	0.5	14.6	157.2	9.8	3.7	4.9	0.8	32.8	161.6	5.3	93.6	7
L	14	3.8	0.5	11.8	116.2	14.8	4.2	6.8	0.9	39.2	176.5	5.6	89.3	5.7
L	14	1.6	0.5	5.8	135.3	7.1	3.3	5.4	0.8	20.6	147.5	4.7	119.7	5.4
L	14	6	0.6	21.6	850.5	14.3	4.9	5.7	1.2	26.5	276.1	5.7	210	6.2
L	14	6.9	0.6	20.8	75.3	18.9	6	4	1.3	38.3	251	4.8	181.2	4.9
L	14	5.2	0.6	21.4	362.6	19.1	4.8	6.8	1.2	45.6	340.4	9.4	190	9.7
L	14	5.1	0.6	19.2	353.6	12.6	4.9	7.9	1.2	38.3	237.7	8.2	152.3	12.2
L	14	3.2	0.5	10.6	387.2	16.6	2.8	2.4	1	32.8	85.5	6.4	82.8	8.6
L	14	4.3	0.5	8.9	336.1	NA	NA	NA	NA	NA	180	7.3	68.8	11.6
L	14	5	0.5	8.1	338.2	12.9	3.1	4.6	1	35.1	171.8	8.7	80	11.3
L	14	2.9	0.5	8.7	219.6	11.2	3.3	3.1	1	31	NA	4.2	69.1	5.7
L	14	10.2	0.7	20.6	766.9	14.1	3.4	3.5	1.3	46.5	254.1	7.8	84.3	7.8
L	14	7.3	0.6	20.8	520.5	14	4.2	2.6	1.2	57.7	228.2	5.4	85.9	15.8
L	14	8.7	0.6	16	271.4	9.2	3.5	4.7	1.2	46.3	204.7	7.3	86.1	7.4
L	14	7.3	0.5	13.1	383	9.3	4.1	3.9	1.1	36.1	156.9	7	90.8	6.8
L	14	4.3	0.6	11.9	211.5	8.2	3.4	2	0.8	31.4	131	5.3	106	8.9
L	14	3.7	0.5	9.6	96.6	9.7	4.1	2.4	0.8	30	117.7	4.4	88	6.4
L	14	3	0.5	8.4	83.8	14.4	4.6	1.7	0.9	30.2	249.4	7.5	113.7	6
L	14	6.8	0.6	18.1	514.8	9.8	2	2	0.8	32	197.7	4.4	94.8	6
L	14	4.3	0.6	15.6	158.6	12.2	5.4	2	1	36.5	300.4	9.7	114.3	10.4
L	14	6.5	0.6	12.7	864.1	12.6	4.4	3.1	1	30	243.1	7.5	105.2	5.7

L	14	4.9	0.5	13.9	316.1	9.4	4.9	1.7	1	33.5	185.9	5.4	98.3	9.8
L	14	7.6	0.6	11.5	195.9	9	3.9	7.2	1	38.6	228.2	7.1	101.5	18.8
VL	14	5.9	0.5	11	105	11.6	4.2	5.4	0.9	32.6	125.5	4.2	81.9	5.5
VL	14	9	0.6	14.7	316.8	11.1	4.3	5.8	0.8	31.4	149.8	5.7	86	6.4
VL	14	2.8	0.6	19.8	776.4	8.9	4.3	2.8	0.9	23.5	69.8	3.9	77.2	4.7
VL	14	2.7	0.6	11	231.6	7.8	4.7	3.1	0.8	29.8	130.2	4	70.9	4.2
VL	14	3.8	0.8	23.7	453.6	12.7	5	5.8	1.1	29.2	160.8	5.8	149.3	4.2
VL	14	4.1	0.6	20.4	252.5	11.5	5.2	4.5	1.1	31	171	7.4	184.4	4.4
VL	14	8.6	0.6	NA	177.5	NA	NA	NA	NA	NA	NA	NA	NA	NA
VL	14	1.5	0.7	21.4	350.4	12.9	6	1.4	1.2	26	NA	NA	NA	NA
VL	14	2.7	0.5	12.9	378.7	9.3	2.5	3.2	1	24.5	127.8	5.7	84.5	6.2
VL	14	7.3	0.5	11.2	848.4	11.1	3.5	2.9	1	31.2	172.6	6.8	66.4	12.8
VL	14	3	0.6	12.7	483	NA	NA	NA	NA	NA	102.8	4.3	69.7	3.4
VL	14	NA	NA	NA	NA	10.5	4	4.1	0.9	29.2	NA	NA	NA	NA
VL	14	5.2	0.7	17.3	558.8	9.5	4.9	3.6	1.1	38.8	235.3	11.5	130	12.6
VL	14	5.2	0.7	17.1	490.9	12.6	5.6	4.1	1.2	35.9	263.5	8.9	87.6	5.7
VL	14	NA	NA	NA	NA	7.7	4.7	3.2	1.2	41.2	190.6	8.6	97.7	6.4
VL	14	NA	NA	NA	NA	8.5	4.8	1.4	1.1	47.1	217.3	6.8	79.5	9.7
VL	14	1.9	0.7	23.9	322.7	9.4	1.8	2	0.8	24.7	222	4.8	97.3	7.3
VL	14	2.5	0.5	15.4	204.1	8.1	3.2	2.8	0.9	25.3	213.3	6.1	96.1	7.4
VL	14	9.6	0.8	14	403.4	8.9	5.2	4.8	1.1	40.6	300.4	6.8	132.8	6.4
VL	14	5.1	0.6	18.1	194.8	10.5	4.7	2.6	1.1	34.1	208.6	7.1	182.4	6.5
VL	14	3.7	0.6	13.6	150.1	NA	NA	NA	NA	30	298.8	7.7	116.5	10.9
VL	14	4.5	0.6	12.9	277.8	9.7	4.2	0.6	1.1	32.9	260.4	6.5	119.3	7.9
H	14	3.2	0.5	8.7	209.3	9.2	4	3.1	0.9	37.7	117.7	5.2	89.8	5
H	14	9.5	0.6	19.3	701.9	7.7	4.2	4.7	1	36.3	146.7	5.4	82.5	5.1
H	14	4.4	0.5	12.1	294.4	9.4	3.3	4.6	0.9	36.7	127.8	6.3	74.8	6

H	14	1.7	0.6	10.2	222.1	10.4	3.5	7.3	0.9	38.4	106.7	4.9	70.5	6.1
H	14	2.9	0.6	15	195	15.1	4.3	4.8	1.1	30.2	171	5.2	204.3	5.2
H	14	2.8	0.5	9.8	89.7	9.5	4.9	3.5	1.2	33.5	267.5	8.2	226.9	14.2
H	14	7.7	0.6	16.7	438.7	9.2	5.3	6.3	1.2	37.5	189	7.5	161.9	6.3
H	14	2.3	0.6	19.6	169.5	10.8	5.5	6.4	1.2	32.5	240.8	7.3	168.4	6.3
H	14	2.6	0.5	6.1	285	11.3	2.8	2.8	1	33.1	178.8	5.6	80.3	8.2
H	14	3.7	0.5	9.4	334.5	11.8	5.5	3.8	1.1	42	105.1	5.4	83.9	7.8
H	14	2.4	0.5	15.6	315.9	11.2	3.9	3.1	1	28	102.8	5.3	89.1	8.6
H	14	3.8	0.5	13.9	321.8	7.1	3.8	3.7	1	30.2	104.3	3.6	91.1	9.7
H	14	4.7	0.6	18.3	324.8	11.1	5.7	3.5	1.2	45	181.2	5.2	88.1	7.8
H	14	5.2	0.6	7.5	331.3	6.3	2.8	2.6	1	19.8	258	7	96.1	10.1
H	14	11.4	0.6	23.9	257.9	9.4	5	1	1.3	48	214.1	7.1	94	7.1
H	14	4.7	0.7	29	373.3	6.9	3.8	3.5	1.1	37.1	282.4	8.9	91.2	8.9
H	14	1.5	0.5	17.3	166.8	5.2	2.6	3.1	1	30	123.1	4.2	81.9	3.5
H	14	3.4	0.5	NA	92.9	13.3	4.1	0.6	1	36.1	167.8	5.2	146.3	7.8
H	14	6.7	0.6	11.5	361.5	9.3	3.5	2	0.9	29.8	171.8	4.8	116.3	6
H	14	3.5	0.5	NA	151.9	8.5	3.5	3.4	0.9	25.9	250.2	5.7	119.2	8.1
H	14	6.7	0.5	10.4	362.9	8.2	5.3	3.9	1	37.7	200.8	3.9	98.1	7.1
H	14	9.2	0.7	32.7	711.9	9.3	4.6	3.4	1.2	37.1	232.9	5.1	116.4	6.8
H	14	6.5	0.7	25.2	214	11.5	5	3.9	1.3	53.5	219.6	7.5	150.5	14.5
H	14	4.5	0.6	20.4	200.1	9.3	5.3	4	1.1	37.3	256.5	7	126.4	7.2
VH	14	2	0.5	16.2	216.7	13.8	4.3	4.2	1.1	33.1	126.3	3.4	77.5	4.9
VH	14	NA	NA	NA	NA	10.8	4.1	2.9	1.1	40.6	69.8	4.5	64.1	10.1
VH	14	6.8	0.5	21	149.7	20.6	4.9	4.6	1.2	52.1	182	5.7	79.3	8.1
VH	14	3.3	0.5	16.7	148.6	10.2	3	4.6	1.1	44	187.5	5.4	66.1	6.5
VH	14	12	0.6	17.5	654.7	12.9	5	5.9	1.3	38.3	251.8	9.4	190.4	6
VH	14	7.2	0.6	13.9	219.5	11.5	5	4.1	1.3	40.2	283.9	7.5	192.8	8.9

VH	14	1.3	0.7	12.7	212.1	8.5	4.1	4.2	1	23.1	251.8	7.1	154.5	7.1
VH	14	4.2	0.5	10.7	195	8.5	4.1	6.2	1.1	30.4	178	6.4	106.7	4.2
VH	14	4.2	0.6	12.9	351	9.6	6.1	3.5	1.2	56.3	137.3	5.5	77.5	7.4
VH	14	0.9	0.5	9.2	422.3	10.2	3.1	2.8	1.1	38.6	164.7	6.8	77.9	17.3
VH	14	3.6	0.5	8.7	339.3	10.8	3.9	4.9	1	39.4	105.9	6.5	83.5	13
VH	14	5	0.6	10.8	406.3	10.3	3.4	4.5	1.1	35.7	98	5.4	77.7	7.9
VH	14	5.9	0.6	16.9	747	9.9	5.7	3.1	1.2	45.7	264.3	9.2	108.4	10.2
VH	14	6.4	0.6	17.7	527	12.2	3.8	3.5	1.2	45.9	222.8	9.4	103.2	9.7
VH	14	2.3	0.6	17.9	594.9	11.1	5.1	2.6	1.3	53.7	316.1	7.7	98.7	6.8
VH	14	5.1	0.6	20.8	263.3	7.7	5.5	4.4	1.2	36.1	284.7	6.7	105.7	8
VH	14	6.3	0.5	14.4	126.4	NA	NA	NA	NA	NA	NA	NA	NA	NA
VH	14	1.8	0.6	10.1	137	11	3.5	3.2	1	46.9	215.7	4.3	91.3	6.8
VH	14	2.7	0.6	14.2	107.6	6.8	5.7	1.2	1.1	30.4	254.9	8.2	132.7	7.8
VH	14	8.5	0.6	13.9	116.1	7.2	3.9	2.7	1.2	36.1	235.3	5.2	134.3	6.9
VH	14	7	0.7	25.8	159.7	11.7	4.2	2.3	1.2	46.1	303.5	7.8	116.3	9.7
VH	14	6.1	0.7	15	739.6	5.3	2.7	1.7	1	32	262.8	7.5	117.6	12.9
