# Supplementary material for: Interactions between ionizing radiation and Vairimorpha (Nosema) ceranae on the honeybee, Apis mellifera L
Source: PLoS One. 2026 Jan 9;21(1):e0339853. doi: 10.1371/journal.pone.0339853 (PMC12788649; doi:10.1371/journal.pone.0339853)
Supplement: S6 Table — C: Control bees, neither irradiated nor infected. V: Bees only infected. H: Bees only irradiated at 14 mGy/h. VH: Bees both infected and irradiated at 14 mGy/h. NA: not available. (PDF) [file pone.0339853.s008.pdf]

S6 Table. Raw data of syrup consumption and mortality from Experiment B. C: Control bees, neither irradiated nor infected. V: Bees only infected. H: Bees only irradiated at 14 mGy/h. VH: Bees both infected and irradiated at 14 mGy/h. NA: not available.

Modality	Days of irradiation	Sirup consumption (µL/bee)	Number of dead bees	Number of live bees
C	0	52.3	0	52
C	0	50.6	0	50
C	0	42.8	0	48
C	0	43	0	50
C	0	56.3	0	49
C	0	56.6	1	51
C	0	55.9	1	45
C	0	56	0	50
C	0	53.7	2	48
C	0	56.8	0	49
C	0	47.7	0	50
C	0	61.3	0	51
V	0	48.8	0	45
V	0	42.5	1	46
V	0	52.4	0	47
V	0	61.2	0	51
V	0	60.4	0	51
V	0	56.6	1	35
V	0	61.4	1	45
V	0	63.5	0	36
V	0	53.9	1	49
V	0	71.2	0	59
V	0	58	0	45
V	0	99.4	0	52
H	0	59.7	0	48
H	0	55.6	0	46
H	0	54.9	0	51
H	0	63.6	0	44
H	0	52.9	1	51
H	0	60.1	1	49
H	0	68.4	0	40
H	0	73.3	0	44
VH	0	64	0	47
VH	0	50.7	0	48
VH	0	48.4	1	49
VH	0	59.9	1	57
VH	0	48.2	0	57

VH	0	55.9	1	49
VH	0	60.9	0	57
VH	0	55	0	52
C	2	74.7	0	52
C	2	64.7	0	50
C	2	108.9	0	48
C	2	76.4	0	50
C	2	68.5	0	49
C	2	71.9	0	52
C	2	60.3	1	45
C	2	59.4	1	49
V	2	100.9	0	45
V	2	95.7	1	46
V	2	67.6	0	47
V	2	80.4	0	51
V	2	74	0	51
V	2	113.6	0	36
V	2	73.1	1	45
V	2	91.9	2	34
H	2	93.1	0	48
H	2	82.1	0	46
H	2	93.6	0	51
H	2	87.3	1	43
H	2	98.2	1	51
H	2	94.1	0	50
H	2	85.4	1	39
H	2	107.2	0	44
VH	2	100.2	0	47
VH	2	82.6	0	48
VH	2	68.7	0	50
VH	2	74.4	1	57
VH	2	75.1	1	56
VH	2	76.4	1	49
VH	2	82.1	0	57
VH	2	64.6	1	51
C	4	110.8	0	48
C	4	75.2	0	50
C	4	77.1	0	49
C	4	70.2	0	52
C	4	81	1	45
C	4	79.1	1	49
V	4	72.4	0	47
V	4	80.2	0	51

V	4	71.5	1	50
V	4	103.2	0	36
V	4	79.4	3	43
V	4	105.4	2	34
H	4	82.9	2	49
H	4	86.8	1	43
H	4	89.6	3	49
H	4	93	1	49
H	4	67.2	3	37
H	4	96.2	2	42
VH	4	81.3	0	50
VH	4	61.9	2	56
VH	4	70.1	1	56
VH	4	80.6	3	47
VH	4	81.9	4	53
VH	4	67.7	1	51
C	6	67.5	1	48
C	6	83.7	1	51
C	6	74.3	2	44
C	6	95.9	1	49
V	6	72.6	2	49
V	6	87.5	1	35
V	6	88.9	7	39
V	6	110.2	3	33
H	6	81.7	4	48
H	6	88.4	3	47
H	6	84.4	3	37
H	6	84.9	2	42
VH	6	84.3	1	56
VH	6	82.9	4	46
VH	6	70.6	7	50
VH	6	65.5	4	48
C	8	60.6	1	48
C	8	71.7	1	51
C	8	69.4	3	43
C	8	81.2	1	49
C	8	72.6	6	45
C	8	79.2	5	31
C	8	68.9	10	36
C	8	80.8	4	32
H	8	69.9	9	43
H	8	72.4	4	46
H	8	81	4	36

H	8	66.1	7	37
VH	8	60	5	52
VH	8	68.9	9	41
VH	8	62	11	46
VH	8	49.4	5	47
C	11	121.5	5	41
C	11	115.9	2	48
V	11	76.1	17	29
V	11	143.2	5	31
H	11	121.8	6	34
H	11	96	10	34
VH	11	118	19	38
VH	11	84.2	12	40
C	14	99.8	11	35
C	14	88.2	4	46
V	14	87.5	21	25
V	14	104.8	9	27
H	14	97.6	8	32
H	14	79.1	13	31
VH	14	94.9	29	28
VH	14	87.6	25	27
