# Supplementary material for: Interactions between ionizing radiation and Vairimorpha (Nosema) ceranae on the honeybee, Apis mellifera L
Source: PLoS One. 2026 Jan 9;21(1):e0339853. doi: 10.1371/journal.pone.0339853 (PMC12788649; doi:10.1371/journal.pone.0339853)
Supplement: S7 Table — C: Control bees, neither irradiated nor infected. V: Bees only infected. H: Bees only irradiated at 14 mGy/h. VH: Bees both infected and irradiated at 14 mGy/h. NA: not available. (PDF) [file pone.0339853.s009.pdf]

S7 Table. Raw data of spore numbers from Experiment B. C: Control bees, neither irradiated nor infected. V: Bees only infected. H: Bees only irradiated at 14 mGy/h. VH: Bees both infected and irradiated at 14 mGy/h. NA: not available.

Modality	Days of irradiation	Spore numbers ($x10^6$ spores/bee)
V	0	0.7
V	0	0.9
V	0	1.3
V	0	1.6
V	0	1.3
V	0	0.8
V	0	0.8
V	0	1.3
V	0	1.6
V	0	1.6
C	2	0.9
C	2	0.8
C	2	1.1
C	2	1
C	2	1
C	2	0.7
C	2	0.6
C	2	0.6
C	2	1
C	2	1.1
V	2	1.5
V	2	1.7
V	2	1.8
V	2	2.5
V	2	2
V	2	1.2
V	2	2.1
V	2	2.2
V	2	2.3
V	2	2.5
H	2	0.6
H	2	0.7
H	2	0.8
H	2	1.3
H	2	0.7
H	2	0.6
H	2	0.8

H	2	0.8
H	2	1.1
H	2	1.1
VH	2	1.7
VH	2	1.3
VH	2	2.1
VH	2	2.4
VH	2	2.2
VH	2	1
VH	2	1.4
VH	2	1.9
VH	2	2
VH	2	1.8
C	4	0.6
C	4	0.7
C	4	1.3
C	4	1
C	4	1.2
C	4	0.5
C	4	0.8
C	4	1.1
C	4	1.5
C	4	1.3
V	4	2.4
V	4	4
V	4	3.8
V	4	3.3
V	4	4.1
V	4	1.7
V	4	3
V	4	2.7
V	4	2.8
V	4	2.6
H	4	0.8
H	4	1.4
H	4	1.4
H	4	1.6
H	4	1.9
H	4	0.5
H	4	0.6
H	4	0.9
H	4	0.8
H	4	0.8

VH	4	1.5
VH	4	1.9
VH	4	2.1
VH	4	1.7
VH	4	2.3
VH	4	2.9
VH	4	2.9
VH	4	3.5
VH	4	3.9
VH	4	4.2
C	8	0.9
C	8	1
C	8	1.7
C	8	1.3
C	8	1.6
C	8	0.7
C	8	0.9
C	8	1.2
C	8	0.7
C	8	0.9
V	8	13.8
V	8	19.1
V	8	18.7
V	8	19.6
V	8	16.3
H	8	1.2
H	8	1
H	8	1.8
H	8	1.3
H	8	1.5
H	8	1
H	8	1.7
H	8	1.8
H	8	1.8
H	8	2
VH	8	12.6
VH	8	17.9
VH	8	17.1
VH	8	17.8
VH	8	16.7
VH	8	9.5
VH	8	14.9
VH	8	15.1

VH	8	13.1
VH	8	14.7
C	14	1
C	14	1.6
C	14	2.3
C	14	2.4
C	14	2.6
C	14	0.8
C	14	1.4
C	14	1.6
C	14	2
C	14	2.1
V	14	13.7
V	14	23.1
V	14	26.1
V	14	24.7
V	14	17
V	14	13.8
V	14	20.3
V	14	20.1
H	14	2
H	14	3.4
H	14	3.4
H	14	3.5
H	14	3.8
H	14	0.8
H	14	1.6
H	14	1.6
H	14	1.7
H	14	1.7
VH	14	21.8
VH	14	34.1
VH	14	34.9
VH	14	33.7
VH	14	19.7
VH	14	30.9
VH	14	37.8
VH	14	38.3
