# Supplementary material for: Interactions between ionizing radiation and Vairimorpha (Nosema) ceranae on the honeybee, Apis mellifera L
Source: PLoS One. 2026 Jan 9;21(1):e0339853. doi: 10.1371/journal.pone.0339853 (PMC12788649; doi:10.1371/journal.pone.0339853)
Supplement: S8 Table — C: Control bees, neither irradiated nor infected. V: Bees only infected. H: Bees only irradiated at 14 mGy/h. VH: Bees both infected and irradiated at 14 mGy/h. NA: not available. (PDF) [file pone.0339853.s010.pdf]

S8 Table. Raw data of biomarkers from Experiment B. C: Control bees, neither irradiated nor infected. V: Bees only infected. H: Bees only irradiated at 14 mGy/h. VH: Bees both infected and irradiated at 14 mGy/h. NA: not available.

Modality	Days of irradiation	Head AChE activity (mAU. $min^{-1}.mg^{-1}$ of tissue)	Head CAT activity (mAU. $min^{-1}.mg^{-1}$ of tissue)	Head GST activity (mAU. $min^{-1}.mg^{-1}$ of tissue)	Head SOD activity (mAU. $min^{-1}.mg^{-1}$ of tissue)	Abdomen ATP activity (LI.mg^{-1} of tissue)	Abdomen Pox activity (mAU. $min^{-1}.mg^{-1}$ of tissue)	Abdomen G6PDH activity (mAU. $min^{-1}.mg^{-1}$ of tissue)	Abdomen GaPDH activity (mAU. $min^{-1}.mg^{-1}$ of tissue)	Abdomen CaE1 activity (mAU.mg^{-1} of tissue)	Abdomen CaE3 activity (mAU. $min^{-1}.mg^{-1}$ of tissue)	Midgut ALP activity (mAU. $min^{-1}.mg^{-1}$ of tissue)
C	0	94	211	75.9	3.3	33.3	9.3	23.9	144	0.3	0.8	6.6
C	0	149.5	241.8	71.8	2.4	29.1	11.3	NA	12.7	0.3	0.7	5.1
C	0	100.7	192.4	68.2	3.1	36.8	9.6	21.6	99.7	NA	0.7	5.1
C	0	44.9	49	55.8	1.6	31.1	13.7	19.5	120.2	0.3	0.8	5.6
C	0	58.8	61.8	64.8	3.6	27.3	12.2	NA	NA	0.3	0.7	4.8
C	0	88.5	62.9	58.8	3.4	46.6	3.8	20.2	112.7	0.3	0.5	3.5
C	0	62.9	64.7	58	3.6	38.9	1.4	9.4	68	0.3	0.6	5.2
C	0	NA	NA	NA	NA	47.2	6.9	10.2	107.3	0.3	0.8	4.1
C	0	NA	NA	NA	NA	29.9	12.4	17.9	130.7	0.3	0.8	9.9
C	0	NA	NA	NA	NA	35	6.8	17.1	101	0.3	0.7	6.8
C	0	NA	NA	NA	NA	36.3	12.7	25.4	137.3	0.5	0.8	7.2
C	0	NA	NA	NA	NA	33.5	11.6	NA	87.3	0.3	0.6	9.1
C	0	NA	NA	NA	NA	29.3	10.3	17.9	114	0.3	0.6	6.3
C	0	NA	NA	NA	NA	45.4	13.5	12.9	39.3	0.3	0.7	8.3
C	0	NA	NA	NA	NA	39.5	6.6	15.1	154	0.3	0.8	4.8
C	0	NA	NA	NA	NA	29.5	9.7	20.7	154	0.3	0.7	8.9
V	0	65.7	65.1	60.3	3	43	11.1	27.5	208	0.3	0.6	11.2
V	0	82.2	57.7	60.6	3.5	34.6	3	9.9	106.7	0.3	0.6	6.6
V	0	62.6	74.9	62.9	4	36.9	7.8	15	112.7	0.3	0.7	4.7
V	0	74.7	73.5	73.4	3.5	43.7	3.2	7.4	57.3	0.3	0.7	2.8
V	0	54.8	52.2	70.1	4	51.5	7.2	12.5	180.7	0.2	0.6	6

V	0	58	34.1	62.9	5.5	37.4	8.7	16.3	84	0.2	0.6	5.3
V	0	102.6	14.1	67.6	3.5	47.3	2.9	10.5	124.7	0.3	0.6	16.6
V	0	53.8	74.1	66	3.2	42	2.4	8.5	68	0.3	0.7	7.6
V	0	117.4	80.8	59.8	3.1	40.1	0.8	11.1	210	0.4	0.8	11.1
V	0	117.7	81.8	60.2	2.2	38.5	6.8	14.7	148	0.3	0.7	5
V	0	109.7	74.5	52.2	2.8	46.6	3	25.4	170	NA	NA	8.9
V	0	81.1	72.9	74.3	3.1	46.9	6.1	14.3	160	NA	NA	7.9
V	0	118.9	65.9	75.4	3.2	NA	NA	NA	NA	NA	NA	NA
V	0	104.2	74.1	67.9	3.5	NA	NA	NA	NA	NA	NA	NA
V	0	70.4	65.5	59	3.8	NA	NA	NA	NA	NA	NA	NA
V	0	78.3	76.5	55.5	1.8	NA	NA	NA	NA	NA	NA	NA
V	0	90.4	51.8	54.1	2.7	NA	NA	NA	NA	NA	NA	NA
H	0	94	211	75.9	3.3	36.8	9.6	21.6	99.7	0.3	0.8	6.6
H	0	149.5	241.8	71.8	2.4	31.1	13.7	19.5	120.2	0.3	0.7	5.1
H	0	100.7	192.4	68.2	3.1	33.3	9.3	23.9	144	NA	0.7	5.1
H	0	44.9	49	55.8	1.6	29.1	11.3	NA	12.7	0.3	0.8	5.6
H	0	58.8	61.8	64.8	3.6	27.3	12.2	NA	NA	0.3	0.7	4.8
H	0	88.5	62.9	58.8	3.4	46.6	3.8	20.2	112.7	0.3	0.5	3.5
H	0	62.9	64.7	58	3.6	38.9	1.4	9.4	68	0.3	0.6	5.2
H	0	NA	NA	NA	NA	47.2	6.9	10.2	107.3	0.3	0.8	4.1
H	0	NA	NA	NA	NA	35	6.8	17.1	101	0.3	0.8	9.9
H	0	NA	NA	NA	NA	36.3	12.7	25.4	137.3	0.3	0.7	6.8
H	0	NA	NA	NA	NA	33.5	11.6	NA	87.3	0.5	0.8	7.2
H	0	NA	NA	NA	NA	29.9	12.4	17.9	130.7	0.3	0.6	9.1
H	0	NA	NA	NA	NA	29.3	10.3	17.9	114	0.3	0.6	6.3
H	0	NA	NA	NA	NA	45.4	13.5	12.9	39.3	0.3	0.7	8.3
H	0	NA	NA	NA	NA	39.5	6.6	15.1	154	NA	NA	4.8

H	0	NA	NA	NA	NA	29.5	9.7	20.7	154	NA	NA	8.9
VH	0	65.7	65.1	60.3	3	43	11.1	27.5	208	0.3	0.8	11.2
VH	0	82.2	57.7	60.6	3.5	34.6	3	9.9	106.7	0.3	0.7	6.6
VH	0	62.6	74.9	62.9	4	36.9	7.8	15	112.7	0.3	0.6	4.7
VH	0	74.7	73.5	73.4	3.5	43.7	3.2	7.4	57.3	0.3	0.6	2.8
VH	0	54.8	52.2	70.1	4	51.5	7.2	12.5	180.7	0.3	0.7	6
VH	0	58	34.1	62.9	5.5	37.4	8.7	16.3	84	0.3	0.7	5.3
VH	0	102.6	14.1	67.6	3.5	47.3	2.9	10.5	124.7	0.2	0.6	16.6
VH	0	53.8	74.1	66	3.2	42	2.4	8.5	68	0.2	0.6	7.6
VH	0	117.4	80.8	59.8	3.1	40.1	0.8	11.1	210	0.3	0.6	11.1
VH	0	117.7	81.8	60.2	2.2	38.5	6.8	14.7	148	0.3	0.7	5
VH	0	109.7	74.5	52.2	2.8	46.6	3	25.4	170	0.4	0.8	8.9
VH	0	81.1	72.9	74.3	3.1	46.9	6.1	14.3	160	0.3	0.7	7.9
VH	0	118.9	65.9	75.4	3.2	NA	NA	NA	NA	NA	NA	NA
VH	0	104.2	74.1	67.9	3.5	NA	NA	NA	NA	NA	NA	NA
VH	0	70.4	65.5	59	3.8	NA	NA	NA	NA	NA	NA	NA
VH	0	78.3	76.5	55.5	1.8	NA	NA	NA	NA	NA	NA	NA
VH	0	90.4	51.8	54.1	2.7	NA	NA	NA	NA	NA	NA	NA
C	2	58.5	28.6	50.7	2.4	61.5	7.6	18.5	164.7	0.5	0.6	10.5
C	2	93.5	44.3	47.8	0.4	32.3	6.8	12.8	75.3	0.5	0.7	10
C	2	89	57.7	54.9	0	39.7	4.8	28.5	242.7	0.4	0.6	12.4
C	2	117.4	74.5	63.2	1.1	29.5	4.8	15.9	200	0.4	0.6	6.5
C	2	62.2	92.6	59.7	3.2	30.3	9.2	25.4	164	0.4	0.6	6
C	2	92.7	54.5	59.2	2.4	22.3	5.6	18.7	74.7	0.5	0.7	5.1
C	2	NA	NA	NA	NA	34.2	3.4	21.9	236.7	0.4	0.7	8.6
C	2	NA	NA	NA	NA	33.9	10.4	23.9	225.3	0.4	0.6	9.1
C	2	NA	NA	NA	NA	64.3	6.6	23	191.3	0.4	0.6	9.9

C	2	NA	NA	NA	NA	46.6	1.9	44.3	235.3	0.5	0.8	11.6
C	2	NA	NA	NA	NA	41.3	6.5	19	262.7	0.4	0.7	8.3
C	2	NA	NA	NA	NA	31.8	6.9	18.9	128	0.4	0.6	7.6
V	2	124.9	24.1	59.8	3.1	29.7	6.4	13.9	130	0.3	0.6	7.9
V	2	121.4	37.3	67.4	3.5	59.9	9.8	22.8	143.3	0.3	0.6	11.5
V	2	111.5	22.4	61.9	2.8	41.4	5.5	19.8	154	0.4	0.6	8
V	2	90.5	25.9	58.1	1.2	27.9	4.6	15.9	178.7	0.2	0.6	7.2
V	2	135.1	25.3	56.5	0	52.1	6.2	12.7	57.3	0.4	0.6	9.2
V	2	95.7	23.5	61.2	1.3	36.7	6.6	28.7	212.7	0.4	0.6	7.6
V	2	NA	NA	NA	NA	41.8	1.7	14.3	150	0.4	0.7	14.9
V	2	NA	NA	NA	NA	41.7	9.7	19.5	92.7	0.3	0.6	7.1
V	2	NA	NA	NA	NA	40.9	5.8	18.7	186	0.4	0.7	11.2
V	2	NA	NA	NA	NA	36.1	6.4	22.7	172.7	0.4	0.7	10.4
V	2	NA	NA	NA	NA	49.2	5.2	15.9	131.3	0.4	0.6	9.4
V	2	NA	NA	NA	NA	27.1	4	17.7	99.3	0.4	0.6	9.9
V	2	NA	NA	NA	NA	NA	NA	NA	220	0.4	0.7	NA
H	2	99.9	83.1	56.7	2.4	38	10.5	23.8	NA	0.4	NA	9.1
H	2	103.8	94.7	62	1	38	10.2	34.2	NA	0.4	NA	7.8
H	2	99.5	93.3	59.1	0.2	43.9	9.5	22.7	NA	0.4	NA	9.4
H	2	94.1	26.7	60.1	2.6	39.5	9.9	20.5	NA	0.4	NA	5.3
H	2	101.7	50.6	63.2	3.5	59.5	9.4	15.5	NA	0.4	NA	8.9
H	2	128.2	83.1	68.9	2.1	46.1	15.2	23.6	NA	0.4	NA	10.8
H	2	NA	NA	NA	NA	42.5	6.4	13	131.3	0.2	0.6	14.5
H	2	NA	NA	NA	NA	34.5	1.7	13.8	52.5	0.3	0.6	14.3
H	2	NA	NA	NA	NA	40.5	4.8	8.9	18.2	0.3	0.6	8.3
H	2	NA	NA	NA	NA	43.1	4.9	13.1	131.3	0.3	0.6	10.1
H	2	NA	NA	NA	NA	69.1	4.6	11.5	62.6	0.3	0.6	8.3

H	2	NA	NA	NA	NA	57.7	2.8	7.5	26.3	0.3	0.6	11.5
VH	2	120.7	66.3	56.8	1.5	59.6	11.1	26.3	259.3	0.5	0.7	6.8
VH	2	113.4	63.9	60.3	1.3	40.9	11.8	30	238	0.4	0.7	12.2
VH	2	97.9	64.1	52.5	2.4	37.7	4.4	20.3	116.7	0.4	0.6	10.4
VH	2	101.9	23.5	54.5	2.3	51.7	11.2	46.4	305.3	0.5	0.8	7.4
VH	2	97.7	5.1	58.6	2.1	57.3	11.5	29	281.3	0.4	0.6	4.8
VH	2	112.6	17.1	57.9	2.4	34.1	11.2	34.3	256.7	0.4	0.7	7.9
VH	2	NA	NA	NA	NA	42.3	12.1	24.7	216.7	0.4	0.7	8.2
VH	2	NA	NA	NA	NA	40.7	5.9	19.6	52.7	0.4	0.6	8.5
VH	2	NA	NA	NA	NA	42.9	8.6	12	21.3	0.5	0.7	5.1
VH	2	NA	NA	NA	NA	88.7	12.7	30.2	170	0.5	NA	8.2
VH	2	NA	NA	NA	NA	47.4	10.9	25	241.3	0.4	NA	4.8
VH	2	NA	NA	NA	NA	45.4	10.5	20.4	170.7	0.4	NA	7.6
C	4	105.9	60	56.4	2.6	46.4	7.2	25.2	136.3	0.3	0.9	6.4
C	4	90.8	55.7	55.7	3.1	54.3	9.9	16.7	123.2	0.3	0.7	5
C	4	40.3	128.6	48.6	2.9	79.7	3.6	10.7	101	0.3	0.6	6.5
C	4	NA	NA	NA	NA	40.7	3.8	9.2	24.2	0.4	0.8	14.8
C	4	NA	NA	NA	NA	53.3	2.9	19.5	145.4	0.2	0.8	5.4
C	4	NA	NA	NA	NA	55.7	9.7	14	54.5	0.3	0.7	8.2
C	4	NA	NA	NA	NA	28.1	7.7	12.3	40.4	0.3	0.7	7.9
C	4	NA	NA	NA	NA	40.7	9.4	18.1	38.4	0.3	0.7	6
C	4	NA	NA	NA	NA	61.1	14.4	19.9	139.4	0.3	0.7	5.2
C	4	NA	NA	NA	NA	51	10.5	28	127.3	0.2	0.8	6.4
C	4	NA	NA	NA	NA	35.1	13.8	16.3	129.3	0.3	0.7	6.6
C	4	NA	NA	NA	NA	40.3	14.4	14.7	129.3	0.3	0.7	6
V	4	57.1	7.7	63.5	4.6	39.5	2.3	6.7	26.3	0.3	0.6	6.2
V	4	46.6	14.9	66.8	4.6	48.9	2.7	14.3	133.3	0.2	0.6	7.3

V	4	52.6	25.3	61.4	4.2	56.9	2.4	7.5	36.4	0.3	0.6	6.5
V	4	50.8	61.8	50.5	2.6	32.7	8.7	11.2	137.4	0.2	0.6	3.9
V	4	NA	NA	NA	NA	56.9	4.3	6.4	94.9	0.2	0.5	7.1
V	4	NA	NA	NA	NA	54.2	5	8.9	117.2	0.3	0.6	10.4
V	4	NA	NA	NA	NA	66.7	8.8	20.6	119.2	0.4	0.7	6
V	4	NA	NA	NA	NA	88.7	7.8	13.7	133.3	0.3	0.7	4.9
V	4	NA	NA	NA	NA	62.7	4.9	22	147.5	0.3	0.7	4.7
V	4	NA	NA	NA	NA	42.1	10.4	17.7	141.4	0.3	0.7	5.1
V	4	NA	NA	NA	NA	38.1	2.2	12.3	119.2	0.3	0.7	5.8
V	4	NA	NA	NA	NA	41.3	5.9	19.9	139.4	0.3	0.7	6.4
H	4	91.8	41.2	50.4	1.3	36.8	2.8	7.2	137.3	0.4	0.5	5.3
H	4	101.9	77.7	56.2	1.3	30.8	5.3	4.5	88	0.5	0.5	8
H	4	49.6	101.6	48.2	1.7	48.3	3	3.4	182	0.4	0.5	7.6
H	4	60.4	92.2	50	4	59.9	2.7	3.9	163.3	0.4	0.5	6.4
H	4	93.8	102.8	53.9	3.1	35.5	8.3	5.4	231.3	0.5	0.5	6
H	4	NA	NA	NA	NA	39.3	3.7	5.1	176.7	0.5	0.5	9.2
H	4	NA	NA	NA	NA	39.8	8.6	8.3	231.3	0.4	0.6	4.9
H	4	NA	NA	NA	NA	44.5	12.2	3.2	162.7	0.4	0.6	7.2
H	4	NA	NA	NA	NA	62.1	6.6	5.6	175.3	0.4	0.5	9.8
H	4	NA	NA	NA	NA	37.6	3.6	6.7	164	0.4	0.5	6.3
H	4	NA	NA	NA	NA	47.9	9.2	12.3	181.3	0.5	0.6	8.8
H	4	NA	NA	NA	NA	43.7	1.6	3	221.3	0.2	0.5	NA
VH	4	103.4	22.8	49.7	2.1	55.7	10.7	17.4	115.1	0.2	0.7	5.7
VH	4	125.8	34.9	52.6	1.7	50.5	3.4	5	28.3	0.3	0.5	4.6
VH	4	112.6	30	47.2	0.7	43.1	6.1	5.9	21.2	0.3	0.7	2.6
VH	4	99.9	20.4	47.2	1.8	56.3	10.3	18.9	135.3	0.3	0.7	6
VH	4	108	7.7	47.1	1.7	55.5	12.6	17.6	131.3	0.4	0.8	3.7

VH	4	105.1	7.8	50.2	1.1	61.2	11.1	17.5	115.1	0.4	0.7	7.3
VH	4	NA	NA	NA	NA	43	4.3	9.1	97	0.3	0.6	4.8
VH	4	NA	NA	NA	NA	47.7	15	11.6	127.3	0.3	0.5	8.9
VH	4	NA	NA	NA	NA	73.5	9.8	17.5	117.2	0.3	0.7	4.2
VH	4	NA	NA	NA	NA	44.3	11.5	13.3	123.2	0.3	0.6	6.4
VH	4	NA	NA	NA	NA	65.2	6.4	11.1	46.5	0.3	0.6	4.9
VH	4	NA	NA	NA	NA	74.8	12.6	15.9	131.3	0.3	0.6	5.6
C	8	105.2	58.8	72.7	2.5	40.3	5.7	23.9	117.3	0.3	0.8	4.9
C	8	103.2	34.7	71.1	2.3	28.3	6.3	14.7	25.3	0.3	0.8	4.2
C	8	107	51.4	74.4	2.3	31.1	5.1	8.2	16.7	0.3	0.8	4.2
C	8	103.1	27.7	67.5	2.5	42.5	3.6	14	34	0.3	0.8	3.8
C	8	58.7	33.7	65.5	3.1	43.5	3.3	14.5	29.3	0.3	0.8	2.7
C	8	57.3	40.8	61.8	2.6	30.6	9.2	11.1	17.3	0.3	0.8	5.3
C	8	NA	NA	NA	NA	58.8	8.1	20.3	188	0.3	0.8	3.8
C	8	NA	NA	NA	NA	66.7	9.7	27.5	230.7	0.3	0.8	5
C	8	NA	NA	NA	NA	95.6	11.4	24.1	186	0.3	0.8	3.8
C	8	NA	NA	NA	NA	65.3	7.2	21.8	46.7	0.3	0.7	4.1
C	8	NA	NA	NA	NA	85.5	5.9	26.7	214.7	0.3	0.9	5.1
C	8	NA	NA	NA	NA	49.2	8	23.4	36.7	0.4	0.8	NA
V	8	90.4	114.5	59.1	3.5	33.6	4.5	5.9	140.7	0.2	0.5	8.8
V	8	97.7	157.3	66.6	3.2	47.8	5.9	7.7	191.3	0.2	0.5	7.4
V	8	86.4	258	69.9	2.9	34.3	5.3	10.1	147.3	0.3	0.5	5.9
V	8	110.1	45.9	66.6	2.1	65.5	7.2	16.6	242.7	0.3	0.6	4.6
V	8	73.3	107.1	59.6	2.4	39.7	4.4	7.3	153	0.3	0.5	6.3
V	8	102.9	82.9	63.8	2.4	47.8	8.9	20.7	309.3	0.3	0.6	4.6
V	8	NA	NA	NA	NA	38.9	1.7	7.1	245.3	0.2	0.5	2.6
V	8	NA	NA	NA	NA	40.9	2.6	3.8	136	0.3	0.5	9.3

V	8	NA	NA	NA	NA	40.3	2	7.1	180	0.3	0.5	3.8
V	8	NA	NA	NA	NA	39.4	7.9	8.9	147.3	0.2	0.5	7.3
V	8	NA	NA	NA	NA	43.7	3.3	9.7	146.7	0.2	0.8	3.9
V	8	NA	NA	NA	NA	42.9	6.3	12.4	264	0.2	0.8	5.2
H	8	47.7	43.1	70	3.3	51.8	2.9	6.9	38.7	0.3	0.4	4.2
H	8	65.7	16.5	66.4	2.8	31.1	5.4	4.7	84.7	0.3	0.4	7.4
H	8	100.9	14.1	72.8	3.6	50.3	2.2	5.4	26.7	0.3	0.5	3.9
H	8	111.8	17.7	69.5	3.1	51.5	3.2	2.9	17.3	0.3	0.5	6
H	8	100	34.1	66.7	3.5	35.2	4.2	9.2	22	0.3	0.5	10
H	8	102.1	11.8	73.3	2	39.8	6.2	8.4	21.3	0.3	0.6	4.9
H	8	NA	NA	NA	NA	32.1	4.5	8.7	28	0.3	0.5	19.4
H	8	NA	NA	NA	NA	29.1	8.8	9	136.7	0.3	0.6	4.7
H	8	NA	NA	NA	NA	42.9	3.7	10.7	188.7	0.3	0.6	4.5
H	8	NA	NA	NA	NA	35.5	4.2	9	148	0.3	0.6	8.6
H	8	NA	NA	NA	NA	41.3	3	5.7	30.7	0.3	0.5	7.3
H	8	NA	NA	NA	NA	32	3.9	4.2	19.3	0.3	0.5	NA
VH	8	134.4	7.1	72.2	2	33.1	3.6	6.9	71	0.4	0.6	4.9
VH	8	81.2	11.4	64.9	2.3	30.9	4.1	7.4	32.7	0.2	0.5	8.3
VH	8	120.3	39.2	69.4	2.7	43.3	4.7	8.1	29.3	0.3	0.6	4.6
VH	8	116.6	49.4	63.7	2.2	40.1	3.7	7.6	28.7	0.3	0.7	4.1
VH	8	108.1	119.6	72	2.4	57	3	5.1	112.7	0.3	0.6	5.7
VH	8	116.7	64.1	71.1	2.1	47.3	3.7	12	188.7	0.3	0.7	5.3
VH	8	NA	NA	NA	NA	31.5	6.9	7.7	25.3	0.3	0.5	5.1
VH	8	NA	NA	NA	NA	37.1	7.2	8.1	122.7	0.3	0.5	4.9
VH	8	NA	NA	NA	NA	77.5	8.4	12.7	175.3	0.3	0.5	3.8
VH	8	NA	NA	NA	NA	106.2	10.6	20.6	263.3	0.3	0.5	4.6
VH	8	NA	NA	NA	NA	37.2	11.3	14	144	0.3	0.4	3

VH	8	NA	NA	NA	NA	119.7	10.8	16.9	159.3	0.3	0.5	NA
C	14	106.6	158.8	63.2	3.1	24.3	9.6	12.9	28	0.3	0.6	5.7
C	14	106.8	75.3	63.1	2.5	24.3	10.2	NA	43.3	0.4	0.6	5.7
C	14	77.7	80.4	57	2.9	23.3	2.9	8.3	28	0.4	0.7	9.6
C	14	94.4	56.1	64.2	3.6	24.5	4.3	10.2	32.7	0.3	0.7	6.1
C	14	52.6	55.3	59.4	4.3	34.6	12.8	12.3	184	0.3	0.6	8.9
C	14	53	195.3	57.9	3.5	26.5	8.9	16.2	51.3	0.3	0.6	5.3
C	14	NA	NA	NA	NA	23.3	14.1	11.5	48.7	0.3	0.7	4.3
C	14	NA	NA	NA	NA	26.1	5.7	15	38	0.4	0.7	6.8
C	14	NA	NA	NA	NA	42.3	12.6	18.3	184.7	0.4	0.6	5.8
C	14	NA	NA	NA	NA	24.9	3.9	10.3	33.3	0.4	0.7	6.3
C	14	NA	NA	NA	NA	28.2	9.2	15.1	184	0.3	0.8	3.5
C	14	NA	NA	NA	NA	NA	1.7	5.7	28	0.3	0.6	NA
V	14	116.7	114.7	74.5	3	32.2	4.5	15.1	214.7	0.3	0.6	9.7
V	14	79.8	88.2	61.9	4.9	23.8	2	7.7	168.7	0.3	0.5	7.3
V	14	70.8	71.8	61.1	3.9	37.5	3.2	8.3	82.7	0.3	0.5	4.9
V	14	31.2	195.7	56.6	3.5	38.7	4	4.1	23.3	0.3	0.5	7.5
V	14	116.9	285.3	62.1	2.5	32.7	4.2	6.9	29.3	0.3	0.6	6
V	14	98.8	201.6	61.2	3.1	NA	NA	NA	13.3	0.2	0.4	4.4
V	14	NA	NA	NA	NA	24.7	6.6	4.8	27.3	0.3	0.5	6.7
V	14	NA	NA	NA	NA	24.5	1.6	7.9	30.7	0.3	0.5	4.9
V	14	NA	NA	NA	NA	24.7	6.1	8.1	38	0.3	0.6	6.2
V	14	NA	NA	NA	NA	23.7	6.3	9.7	27.3	0.3	0.6	7.6
V	14	NA	NA	NA	NA	22.5	6.8	9.5	49.3	0.4	0.8	NA
V	14	NA	NA	NA	NA	23.7	8	9.7	68	0.3	0.5	NA
H	14	42.8	129.4	53	3.8	37.4	2	4.7	24.7	0.3	0.5	11
H	14	86.1	247.7	61.3	3.4	31.9	1.4	5.2	138.7	0.3	0.5	8.5

H	14	88.8	182.4	70	2.9	45.1	1.3	6.7	17.3	0.3	0.5	3.9
H	14	81.4	186.3	68.9	4.2	31.9	4.9	7.7	19.3	0.3	0.5	4.9
H	14	84.7	146.3	71.8	4.2	38.1	4.3	7.6	24.7	0.3	0.6	9.8
H	14	96.5	300	75.8	3.4	44.7	1.1	3.8	30.7	0.3	0.5	7
H	14	NA	NA	NA	NA	34	3.5	7.7	20.7	0.3	0.6	7.9
H	14	NA	NA	NA	NA	33.2	2.8	7.3	25.3	0.3	0.5	4.6
H	14	NA	NA	NA	NA	39.5	8.1	10.3	192.7	0.3	0.6	7.5
H	14	NA	NA	NA	NA	35.1	5.5	6.1	24.7	0.4	0.7	6.7
H	14	NA	NA	NA	NA	40.1	1.7	5.7	23.3	0.3	0.5	9.6
H	14	NA	NA	NA	NA	29	3.8	11.7	20.7	0.4	0.7	8.2
VH	14	77.8	241.6	56.6	3.4	47.4	2	6.6	24.7	0.3	0.5	13.4
VH	14	129.9	163.5	72.3	2.8	46.1	1.3	6.8	43.3	0.5	0.5	8.6
VH	14	110.8	130.6	62.4	3.5	40.8	10.9	11.9	30.7	0.4	0.5	8.8
VH	14	97.9	144.7	65.2	2.7	39.6	7.3	10.1	30	0.5	0.5	7.1
VH	14	100.6	124.7	62.4	2.7	59.1	2.7	6.1	30	0.6	0.5	6.4
VH	14	92.9	77.7	63.9	2.7	36.9	4.3	7.2	26.7	0.5	0.5	6
VH	14	NA	NA	NA	NA	37.4	2.2	5.5	32	0.7	0.5	9.5
VH	14	NA	NA	NA	NA	41.3	7.7	8.3	19.3	0.8	0.5	6.2
VH	14	NA	NA	NA	NA	45.5	6.5	6.7	82	0.7	0.5	5.4
VH	14	NA	NA	NA	NA	40.8	3.6	7.7	20.7	0.4	0.6	7
VH	14	NA	NA	NA	NA	37.9	3.4	6.4	23.3	0.5	0.6	7.8
VH	14	NA	NA	NA	NA	NA	NA	NA	NA	0.4	0.6	6.6
